# Linear assembly of a human centromere on the Y chromosome

**DOI:** 10.1038/nbt.4109

**Published:** 2018-03-19

**Authors:** Miten Jain, Hugh E Olsen, Daniel J Turner, David Stoddart, Kira V Bulazel, Benedict Paten, David Haussler, Huntington F Willard, Mark Akeson, Karen H Miga

**Affiliations:** 1 UC Santa Cruz Genomics Institute, University of California, Santa Cruz, California USA; 2 Oxford Nanopore Technologies, Oxford, UK; 3 Duke Institute for Genome Sciences and Policy, Duke University, Durham, North Carolina USA; 4 Geisinger National, Bethesda, Maryland USA

**Keywords:** Genomics, Centromeres, DNA sequencing, Genome

## Abstract

The human genome reference sequence remains incomplete owing to the challenge of assembling long tracts of near-identical tandem repeats in centromeres. We implemented a nanopore sequencing strategy to generate high-quality reads that span hundreds of kilobases of highly repetitive DNA in a human Y chromosome centromere. Combining these data with short-read variant validation, we assembled and characterized the centromeric region of a human Y chromosome.

**Supplementary information:**

The online version of this article (doi:10.1038/nbt.4109) contains supplementary material, which is available to authorized users.

## Abstract (web summary)

Repeat regions in the human Y chromosome centromere are assembled using long-read nanopore sequencing.

**Supplementary information:**

The online version of this article (doi:10.1038/nbt.4109) contains supplementary material, which is available to authorized users.

## Main

Centromeres facilitate spindle attachment and ensure proper chromosome segregation during cell division. Normal human centromeres are enriched with AT-rich ∼171-bp tandem repeats known as alpha satellite DNA^1^. Most alpha satellite DNAs are organized into higher order repeats (HORs), in which chromosome-specific alpha satellite repeat units, or monomers, are reiterated as a single repeat structure hundreds or thousands of times with high (>99%) sequence conservation to form extensive arrays^2^. Characterizing both the sequence composition of individual HOR structures and the extent of repeat variation is crucial to understanding kinetochore assembly and centromere identity^3,4,5^. However, no sequencing technology (including single-molecule real-time (SMRT) sequencing or synthetic long-read technologies) or a combination of sequencing technologies has been able to assemble centromeric regions because extremely high-quality, long reads are needed to confidently traverse low-copy sequence variants. As a result, human centromeric regions remain absent from even the most complete chromosome assemblies.

Here we apply nanopore long-read sequencing to produce high-quality reads that span hundreds of kilobases of highly repetitive DNA (Supplementary Fig. 1). We focus on the haploid satellite array present on the Y centromere (DYZ3), as it is particularly suitable for assembly owing to its tractable size, well-characterized HOR structure, and previous physical mapping data^6,7,8^.

**Figure 1:**
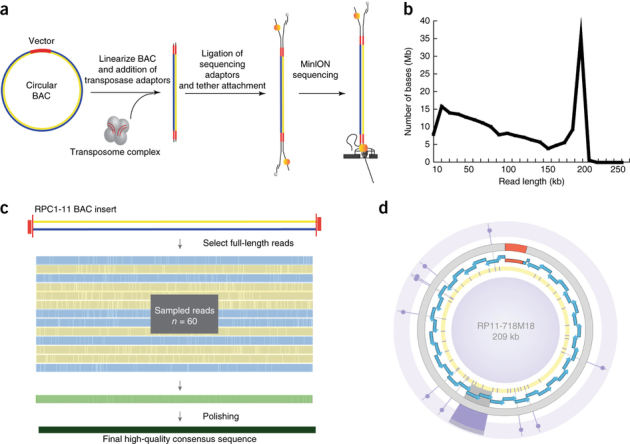
BAC-based longboard nanopore sequencing strategy on the MinION. (**a**) Optimized strategy to cut each circular BAC once with transposase results in a linear and complete DNA fragment of the BAC for nanopore sequencing. (**b**) Yield plot of BAC DNA (RP11-648J18). (**c**) High-quality BAC consensus sequences were generated by multiple alignment of 60 full-length 1D reads (shown as blue and yellow for both orientations), sampled at random with ten iterations, followed by polishing steps (green) with the entire nanopore long-read data and Illumina data. (**d**) Circos representation^20^ of the polished RP11-718M18 BAC consensus sequence. Blue arrowheads indicate the position and orientation of HORs. Purple tiles in yellow background mark the position of the Illumina-validated variants. Additional purple highlight extending from select Illumina-validated variants are used to identify single-nucleotide-sequence variants and mark the site of the DYZ3 repeat structural variants (6 kb) in tandem.

We devised a transposase-based method that we named 'longboard strategy' to produce high-read coverage of full-length bacterial artificial chromosome (BAC) DNA with nanopore sequencing (MinION sequencing device, Mk1B, Oxford Nanopore Technologies). In our longboard strategy, we linearize the circular BAC with a single cut site, then add sequencing adaptors (Fig. 1a). The BAC DNA passes through the pore, resulting in complete, end-to-end sequence coverage of the entire insert. Plots of read length versus megabase yield revealed an increase in megabase yield for full-length BAC DNA sequences (Fig. 1b and Supplementary Fig. 2). We present more than 3,500 full-length '1D' reads (that is, one strand of the DNA is sequenced) from ten BACs (two control BACs from Xq24 and Yp11.2; eight BACs in the DYZ3 locus^9^; Supplementary Table 1).

Correct assembly across the centromeric locus requires overlap among a few sequence variants, meaning that accuracy of base-calls is important. Individual reads (MinION R9.4 chemistry, Albacore v1.1.1) provide insufficient sequence identity (median alignment identity of 84.8% for control BAC, RP11-482A22 reads) to ensure correct repeat assembly^10^. To improve overall base quality, we produced a consensus sequence from 10 iterations of 60 randomly sampled alignments of full-length 1D reads that spanned the full insert length for each BAC (Fig. 1c). To polish sequences, we realigned full-length nanopore reads to each BAC-derived consensus (99.2% observed for control BAC, RP11-482A22; and an observed range of 99.4–99.8% for vector sequences in DYZ3-containing BACs). To provide a truth set of array sequence variants and to evaluate any inherent nanopore sequence biases, we used Illumina BAC resequencing (Online Methods). We used eight BAC-polished sequences (e.g., 209 kb for RP11-718M18; Fig. 1d) to guide the ordered assembly of BACs from p-arm to q-arm, which includes an entire Y centromere.

**Figure 2:**
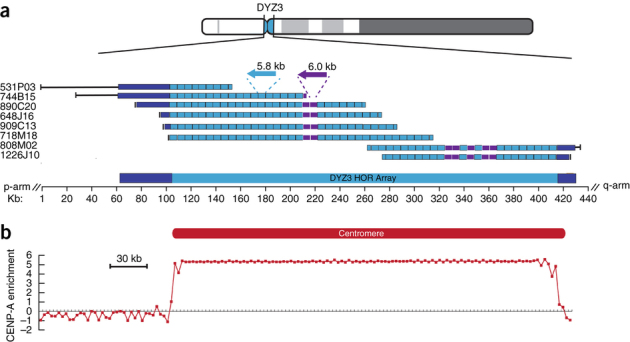
Linear assembly of the RP11 Y centromere. (**a**) Ordering of nine DYZ3-containing BACs spanning from proximal p-arm to proximal q-arm. The majority of the centromeric locus is defined by the DYZ3 conical 5.8-kb HOR (light blue). Highly divergent monomeric alpha satellite is indicated in dark blue. HOR variants (6.0 kb) indicated in purple. (**b**) The genomic location of the functional Y centromere is defined by the enrichment of centromere protein A (CENP-A), where enrichment (∼5–6×) is attributed predominantly to the DYZ3 HOR array.

We ordered the DYZ3-containing BACs using 16 Illumina-validated HOR variants, resulting in 365 kb of assembled alpha satellite DNA (Fig. 2a and Supplementary Data 1). The centromeric locus contains a 301-kb array that is composed of the DYZ3 HOR, with a 5.8-kb consensus sequence, repeated in a head-to-tail orientation without repeat inversions or transposable element interruptions^6,11,12^. The assembled length of the RP11 DYZ3 array is consistent with estimates for 96 individuals from the same Y haplogroup (R1b) (Supplementary Fig. 3; mean: 315 kb; median: 350 kb)^13,14^. This finding is in agreement with pulsed-field gel electrophoresis (PFGE) DYZ3 size estimates from previous physical maps, and from a Y-haplogroup matched cell line (Supplementary Fig. 4).

Pairwise comparisons among the 52 HORs in the assembled DYZ3 array revealed limited sequence divergence between copies (mean 99.7% pairwise identity). In agreement with a previous assessment of sequence variation within the DYZ3 array^6^, we detected instances of a 6.0-kb HOR structural variant and provide evidence for seven copies within the RP11 DYZ3 array that were present in two clusters separated by 110 kb, as roughly predicted by previous restriction map estimates^8^. Sequence characterization of the DYZ3 array revealed nine HOR haplotypes, defined by linkage between variant bases that are frequent in the array (Supplementary Fig. 5). These HOR haplotypes were organized into three local blocks that were enriched for distinct haplotype groups, consistent with previous demonstrations of short-range homogenization of satellite-DNA-sequence variants^6,15,16^.

Functional centromeres are defined by the presence of inner centromere proteins that epigenetically mark the site of kinetochore assembly^17,18,19^. To define the genomic position of the functional centromere on the Y chromosome, we examined the enrichment profiles of inner kinetochore centromere protein A (CENP-A), a histone H3 variant that replaces histone H3 in centromeric nucleosomes, using a Y-haplogroup-matched cell line that offers a similar DYZ3 array sequence (Fig. 2b and Supplementary Data 2)^5,14,19^. We found that CENP-A enrichment was predominantly restricted to the canonical DYZ3 HOR array, although we did identify reduced centromere protein enrichment extending up to 20 kb into flanking divergent alpha satellite on both the p-arm and q-arm side. Thus, we provide a complete genomic definition of a human centromere, which may help to advance sequence-based studies of centromere identity and function.

We applied a long-read strategy to map, sequence, and assemble tandemly repeated satellite DNAs and resolve, for the first time to our knowledge, the array repeat organization and structure in a human centromere. Previous modeled satellite arrays^14^ are based on incomplete and gapped maps, and do not present complete assembly data across the full array. Our complete assembly enables the precise number of repeats in an array to be robustly measured and resolves the order, orientation, and density of both repeat-length variants across the full extent of the array. This work could potentially advance studies of centromere evolution and function and may aid ongoing efforts to complete the human genome.

## Methods

### BAC DNA preparation and validation.

Clones of bacterial artificial chromosomes (BACs) used in this study were obtained from BACPAC RPC1-11 library, Children's Hospital Oakland Research Institute in Oakland, California, USA (https://bacpacresources.org/). BACs that span the human Y centromere, RP11-108I14, RP11-1226J10, RP11-808M02, RP11-531P03, RP11-909C13, RP11-890C20, RP11-744B15, RP11-648J16, RP11-718M18, and RP11-482A22, were determined based on previous hybridization with DYZ3-specific probes, and confirmed by PCR with STSs sY715 and sY78 (ref. 9). Notably, DYZ3 sequences, unlike shorter satellite DNAs, have been observed to be stable and cloned without bias^5,21^. The RP11-482A22 BAC was selected as our control since it had previously been characterized by nanopore long-read sequencing^22^, and presented ∼134 kb of assembled, unique sequence present in the GRCh38 reference assembly to evaluate our alignment and polishing strategy. BAC DNA was prepared using the QIAGEN Large-Construct Kit (Cat No./ID: 12462). To ensure removal of the *Escherichia coli* genome, it was important to include an exonuclease incubation step at 37 °C for 1 h, as provided within the QIAGEN Large-Construct Kit. BAC DNAs were hydrated in TE buffer. BAC Insert length estimates were determined by pulsed-field gel electrophoresis (PFGE) (data not shown).

### Longboard MinION protocol.

MinIONs can process long fragments, as has been previously documented^22^. While these long reads demonstrate the processivity of nanopore sequencing, they offer insufficient coverage to resolve complex, repeat-rich regions. To systematically enrich for the number of long reads per MinION sequencing run, we developed a strategy that uses the Oxford Nanopore Technologies (ONT) Rapid Sequencing Kit (RAD002). We performed a titration between the transposase from this kit (RAD002) and circular BAC DNA. This was done to achieve conditions that would optimize the probability of individual circular BAC fragments being cut by the transposase only once. To this end, we diluted the 'live' transposase from the RAD002 kit with the 'dead' transposase provided by ONT. For PFGE-based tests, we used 1 μl of 'live' transposase and 1.5 μl of 'dead' transposase per 200 ng of DNA in a 10-μl reaction volume. This reaction mix was then incubated at 30 °C for 1 min and 75 °C for 1 min, followed by PFGE. Our PFGE tests used 1% high-melting agarose gels and were run with standard 180° field inversion gel electrophoresis (FIGE) conditions for 3.5 h. An example PFGE gel is shown in Supplementary Figure 6.

For MinION sequencing library preparation, we used 1.5 μl of 'live' transposase and 1 μl of 'dead' transposase (supplied by ONT) per 1 μg of DNA in a 10-μl reaction volume. Briefly, this reaction mix was then incubated at 30 °C for 1 min and 75 °C for 1 min. We then added 1 μl of the sequencing adaptor and 1 μl of Blunt/TA Ligase Master Mix (New England BioLabs) and incubated the reaction for 5 min. This was the adapted BAC DNA library for the MinION. R9.4 SpotON flow cells were primed using the protocol recommended by ONT. We prepared 1 ml of priming buffer with 500 μl running buffer (RBF) and 500 μl water. Flow cells were primed with 800 μl priming buffer via the side loading port. We waited for 5 min to ensure initial buffering before loading the remaining 200 μl of priming buffer via the side loading port but with the SpotON open. We next added 35 μl RBF and 28 μl water to the 12 μl library for a total volume of 75 μl. We loaded this library on the flow cell via the SpotON port and proceeded to start a 48 h MinION run.

When a nanopore run is underway, the amplifiers controlling individual pores can alter voltage to get rid of unadapted molecules that can otherwise block the pore. With R9.4 chemistry, ONT introduced global flicking that reversed the potential every 10 min by default to clear all nanopores of all molecules. At 450 b.p.s., a 200 kb BAC would take around 7.5 min to be processed. To ensure sufficient time for capturing BAC molecules on the MinION, we changed the global flicking time period to 30 min. This is no longer the case with an update to ONT's MinKNOW software, and on the later BAC sequencing runs we did not change any parameters. We acknowledge that generating long (>100 kb) reads presents challenges, given the dynamics of high-molecular-weight (HMW) DNA for ligation, chemistry updates, and delivery of free ends to the pore, reducing the effective yield. We found that high-quality and a large quantity of starting material (i.e., our strategy is designed for 1 μg of starting material that does not show signs of DNA shearing and/or degradation when evaluated by PFGE) and reduction of smaller DNA fragments were necessary for the longboard strategy.

### Protocol to improve long-read sequence by consensus and polishing.

BAC-based assembly across the DYZ3 locus requires overlap among a few informative sequence variants, thus placing great importance on the accuracy of base-calls. Therefore, we employed the following strategy to improve overall base quality. First, we derived a consensus from multiple alignments of 1D reads that span the full insert length for each BAC. Further, polishing steps were performed using realignment of all full-length nanopore reads for each BAC. As a result, each BAC sequencing project resulted in a single, polished BAC consensus sequence. To validate single-copy variants, useful in an overlap-layout-assembly strategy, we included Illumina data sets for each BAC. Illumina data were not used to correct or validate variants observed multiple times within a given BAC sequence due to the reduced mapping quality.

### MinION base-calling.

All of the BAC runs were initially base-called using Metrichor, ONT's cloud basecaller. Metrichor classified reads as pass or fail using a Q-value threshold. We selected the full-length BAC reads from the pass reads. We later base-called all of the BAC runs again using Albacore 1.1.1, which included significant improvements on homopolymer calls. This version of Albacore did not contain a pass/fail cutoff. We reperformed the informatics using Albacore base-calls for full-length reads selected from the pass Metrichor base-calls. We selected BAC full-length reads as determined by observed enrichment in our yield plots (shown in Supplementary Fig. 7 the read versus read length plots converted to yield plots to identify BAC length min-max selection thresholds).

Full-length reads used in this study were determined to contain at least 3 kb of vector sequence, as determined by BLASR^23^ (*-sdpTupleSize 8 -bestn 1 -nproc 8 -m 0*) alignment with the pBACe3.6 vector (GenBank Accession: U80929.2). Reads were converted to the forward strand. Reads were reoriented relative to a fixed 3-kb vector sequence, aligning the transition from vector to insert.

### Derive BAC consensus sequence.

Reoriented reads were sampled at random (blasr_output.py). Multiple sequence alignment (MSA) was performed using kalign^24^. We determined empirically that sampling greater than 60 reads provided limited benefit to consensus base quality (Supplementary Fig. 8). We computed the consensus from the MSA whereas the most prevalent base at each position was called. Gaps were only considered in the consensus if the second most frequent nucleotide at that position was present in less than ten reads. We performed random sampling followed by MSA iteratively 10×, resulting in a panel of ten consensus sequences, observed to provide a ∼1% boost in consensus sequence identity (Supplementary Fig. 8). To improve the final consensus sequence, we next performed a final MSA on the collection of ten consensus sequences derived from sampling.

### Polish BAC consensus sequence.

Consensus sequence polishing was performed by aligning full-length 1D nanopore reads for each BAC to the consensus (BLASR^25^, *-sdpTupleSize 8 -bestn 1 -nproc 8 -m 0*). We used pysamstats (https://github.com/alimanfoo/pysamstats) to identify read support for each base call. We determined the average base coverage for each back, and filtered those bases that had low-coverage support (defined as having less than half of the average base coverage). Bases were lower-case masked if they were supported by sufficient sequence coverage, yet had <50% support for a given base call in the reads aligned.

### Variant validation.

We performed Illumina resequencing (MiSeq V3 600bp; 2 × 300 bp) for all nine DYZ3-containing BACs to validate single-copy DYZ3 HOR variants in the nanopore consensus sequence. Inherent sequence bias is expected in nanopore sequencing^22^, therefore we first used the Illumina matched data sets to evaluate the extent and type of sequence bias in our initial read sets, and our final polished consensus sequence. Changes in ionic current, as individual DNA strands are read through the nanopore, are each associated with a unique 5-nucleotide k-mer. Therefore in an effort to detect inherent sequence errors due to nanopore sensing, we compared counts of 5-mers. Alignment of full-length HORs within each polished BAC sequence to the canonical DYZ3 repeat demonstrated that these sequences are nearly identical, where in RP11-718M18 we detected 1,449 variant positions (42% mismatches, 27% deletions, and 31% insertions) across 202,582 bp of repeats (99.5% identity). Although the 5-mer frequency profiles between the two data sets were largely concordant (Supplementary Fig. 9), we found that poly(dA) and poly(dT) homopolymers were overrepresented in our initial nanopore read data sets, a finding that is consistent with genome-wide observations. These poly(dA) and poly(dT) over-representations were reduced in our quality-corrected consensus sequences especially for 6-mers and 7-mers.

### K-mer method.

Using a k-mer strategy (where *k* = 21 bp), we identified exact matches between the Illumina and each BAC consensus sequence. Illumina read data and the BAC-polished consensus sequences were reformatted into respective k-mer library (where *k* = 21 bp, with 1 bp slide using Jellyfish v2 software^25^), in forward and reverse orientation. K-mers that matched the pBACe3.6 sequence exactly were labeled as 'vector'. K-mers that matched the DYZ3 consensus sequence exactly^14^ were labeled as 'ceny'. We first demonstrated that the labeled k-mers were useful in predicting copy number. Initially, we showed how the ceny k-mer frequency in the BACs predicted the DYZ3 copy number, relative to the number observed in our nanopore consensus (Supplementary Fig. 10a). DYZ3 copy number in each consensus sequence derived from nanopore reads was determined using HMMER3 (ref. 26) (v3.1b2) with a profile constructed from the DYZ3 reference repeat. By plotting the distribution of vector k-mer counts (Supplementary Fig. 10b for RP11-718M18), we observed a range of expected k-mer counts for single-copy sites. DYZ3 repeat variants (single-copy satVARs) were determined as k-mers that (1) did not have an exact match with either the vector or DYZ3 reference repeat, (2) spanned a single DYZ3-assigned variant in reference-polished consensus sequence (i.e., that particular k-mer was observed only once in the reference), (3) and had a k-mer depth profile in the range of the corresponding BAC vector k-mer distribution. As a final conservative measure, satVARs used in overlap-layout-consensus assembly were supported by two or more overlapping Illumina k-mers (Supplementary Fig. 10). To test if it was possible to predict a single-copy DYZ3 repeat variant by chance, or by error introduced in the Illumina read sequences, we ran 1,000 simulated trials using our RP11-718M18 Illumina data. Here, we randomly introduced a single variant into the polished RP11-718M18 DYZ3 array (false positive). We generated 1,000 simulated sequences, each containing a single randomly introduced single-copy variant. Next, we queried if the 21-mer spanning the introduced variant was (a) found in the corresponding Illumina data set and (b) if so, we monitored the coverage. Ultimately, none of the simulated false-positive variants (21-mer) met our criteria of a true variant. That is, although the simulated variants were identified in our Illumina data, they had insufficient sequence coverage to be included in our study. Greater than 95% of the introduced false variants had ≤100× coverage, with only one variant observed to have the maximum value of 300×. True variants were determined using this data set with values from 1,100–1600×, as observed in our vector distribution.

### Alignment method.

We employed a short-read alignment strategy to validate single-copy variants in our polished consensus sequence. Illumina-merged reads (PEAR, standard parameters^27^) were mapped to the RP11 Y-assembled sequence using BWA-MEM^28^. BWA-MEM is a component of the BWA package and was chosen because of its speed and ubiquitous use in sequence mapping and analysis pipelines. Aside from the difficulties of mapping the ultra-long reads unique to this work, any other mapper could be used instead. This involves mapping Illumina data to each BAC consensus sequence. After filtering those alignments with mapping quality less than 20, single-nucleotide DYZ3 variants (i.e., a variant that is observed uniquely, or once in a DYZ3 HOR in a given BAC) were considered “validated” if they had support of at least 80% of the reads and had sequence coverage within the read depth distribution observed in the single-copy vector sequence for each BAC data set.

To explore Illumina sequence coverage necessary for our consensus polishing strategy we initially investigated a range (20–100×) of simulated sequence coverage relative to a 73-kb control region (hg38 chrY:10137141–10210167) within the RP11-531P03 BAC data. Simulated paired read data using the ART Illumina simulator software^29^ was specified for the MiSeq sequencing system (MiSeq v3 (250 bp), or 'MSv3′), with a mean size of 400 bp DNA fragments for paired-end simulations. Using our polishing protocol, where reads are filtered by mapping quality score (i.e., at least a score of 20: that the probability of correctly mapping is log_10_ of 0.01 * -10, or 0.99), base frequency was next determined for each position using pysamstats, and a final, polished consensus was determined by taking the base call at any given position that is represented by sufficient coverage (at least half of the determined average across the entire BAC) and is supported by a percentage of Illumina reads mapped to that location (in our study, we required at least 80%). If we require at least 80% of mapped reads to support a given base call, we determine that 30× coverage is sufficient to reach 99% sequence identity (or the same as our observed identity using our entire Illumina read data set, indicated as a gray dotted line in Supplementary Fig. 11). If we require at least 90% of mapped reads to support a particular variant it is necessary to increase coverage to 70× to reach an equivalent polished percent identity.

To evaluate our mapping strategy, we performed a basic simulation using an artificially generated array of ten identical DYZ3 (5.7 kb) repeats. We then randomly introduced a single base change resulting in a new sequence with nine identical DYZ3 repeats and one repeat distinguished by a single-nucleotide change (Supplementary Fig. 12). We first demonstrate that we are able to confidently detect the single variant by simulating reads from the reference sequence containing the introduced variant of varying coverage and Illumina substitution error rate. Additionally, we investigated whether we would detect the variant as an artifact due to Illumina read errors. To test this, we next simulated Illumina reads from a DYZ3 reference array that did not contain the introduced variant (i.e., ten exact copies of the DYZ3 repeat). We performed this simulation 100×, thus creating 100× reference arrays each with a randomly placed single variant. Within each evaluation we mapped in parallel simulated Illumina reads from (a) the array containing introduced variant sequence and (b) the array that lacked the variant. In experiments where reads containing the introduced variant were mapped to the reference containing the variant, we observed the introduced base across variations of sequence coverage and increased error rates. To validate a variant as “true,” we next evaluated the supporting sequence coverage. For example in 100× coverage, using the default Illumina error rate we observed 96 “true” calls out of 100 simulations, where in each case we set a threshold such that at least 80% of reads that spanned the introduced variant supported the base call. We found that Illumina quality did influence our ability to confidently validate array variants by reducing the coverage. When the substitution error was increased by 1/10th we observed a decrease to only 75 “true” variant calls out of 100× simulations. Therefore, we suspect that Illumina sequencing errors may challenge our ability to completely detect true-positive variants.

In our alternate experiments, although simulated Illumina reads from ten identical copies of the DYZ3 repeat were mapped to a reference containing an introduced variant, we did not observe a single simulation and/or condition with sufficient coverage for “true” validation. We do report an increase in the percentage of reads that support the introduced variant as we increase the Illumina substitution error rate, however, the range of read depth observed across all experiments was far below our coverage threshold. We obtained similar results when we repeated this simulation using sequences from the RP11-718M18 DYZ3 array.

Finally, standard quality Illumina-based polishing with pilon 21 was applied strictly to unique (non-satellite DNA) sequences on the proximal p and q arms to improve final quality. Alignment of polished consensus sequences from our control BAC from Xq24 (RP11-482A22) and non-satellite DNA in the p-arm adjacent to the centromere (Yp11.2, RP11-531P03) revealed base-quality improvement to >99% identity.

### Prediction and validation of DYZ3 array.

BAC ordering was determined using overlapping informative single-nucleotide variants (including the nine DYZ3 6.0 kb structural variants) in addition to alignments directly to either assembled sequence on the p-arm or q-arm of the human reference assembly (GRCh38). Notably, physical mapping data were not needed in advance to guide our assembly. Rather these data were provided to evaluate our final array length predictions. Full-length DYZ3 HORs (ordered 1–52) were evaluated by MSA (using kalign^24^) between overlapping BACs, with emphasis on repeats 28–35 that define the overlap between BACs anchored to the p-arm or q-arm (Supplementary Fig. 13). RPC1-11 BAC library has been previously referenced as derived from a known carrier of haplogroup R1b^30,31^. We compared our predicted DYZ3 array length with 93 R1b Y-haplogroup-matched individuals by intersecting previously published DYZ3 array length estimates for 1000 Genome phase 1 data^13,14^ with donor-matched Y-haplogroup information^32^. To investigate the concordancy of our array prediction with previous physical maps of the Y-centromere we identified the positions of referenced restriction sites that directly flank the DYZ3 array in the human chromosome Y assembly (GRCh38)^6,7,33^. It is unknown if previously published individuals are from the same population cohort as the RPC1-11 donor genome, therefore we performed similar PFGE DYZ3 array PFGE length estimates using the HuRef B-lymphoblast cell line (available from Coriell Institute as GM25430), previously characterized to be in the R1-b Y-haplogroup^34^.

### PFGE alpha satellite Southern.

High-molecular-weight HuRef genomic DNA was resuspended in agarose plugs using 5 × 10^6^ cells per 100 μL 0.75% CleanCut Agarose (CHEF Genomic DNA Plug Kits Cat #: 170-3591 BIORAD). A female lymphoblastoid cell line (GM12708) was included as a negative control. Agarose plug digests were performed overnight (8–12 h) with 30–50 U of each enzyme with matched NEB buffer. PFGE Southern experiments used 1/4–1/2 agarose plug per lane (∼5–10 μg) in an 1% SeaKem LE Agarose gel and 0.5× TBE. CHEF Mapper conditions were optimized to resolve 0.1–2.0 Mb DNAs: voltage 6V/cm, runtime: 26:40 h, in angle: 120, initial switch time: 6.75 s, final switch time: 1 m 33.69 s, with a linear ramping factor. We used the Lambda (NEB; N0340S) and *Saccharomyces cerevisiae* (NEB; N0345S) as markers. Methods of transfer to nylon filters, prehybridization, and chromosome-specific hybridization with 32P-labeled satellite probes have been described^35^. Briefly, DNA was transferred to nylon membrane (Zeta Probe GT nylon membrane; CAT# 162-0196) for ∼24 h. DYZ3 probe (50 ng DNA labeled ∼2 c.p.m./mL; amplicon product using previously published STS DYZ3 Y-A and Y-B primers^36^) was hybridized for 16 h at 42 °C. In addition to standard wash conditions^35^, we performed two additional stringent wash (buffer: 0.1% SDS and 0.1× SSC) steps for 10 min at 72 °C to remove non-specific binding. Image was recovered after 20 h exposure.

### Sequence characterization of Y centromeric region.

The DYZ3 HOR sequence and chromosomal location of the active centromere on the human chromosome Y is not shared among closely related great apes^37^. However, previous evolutionary dating of specific transposable element subfamilies (notably, L1PA3 9.2–15.8 MYA^38^) within the divergent satellite DNAs, as well as shared synteny of 11.9 kb of alpha satellite DNA in the chimpanzee genome Yq assembly indicate that the locus was present in the last common ancestor with chimpanzee (Supplementary Fig. 14).

Comparative genomic analysis between human and chimpanzee were performed using UCSC Genome Browser liftOver^39^ between human (GRCh38, or hg38 chrY:10,203,170–10,214,883) and the chimpanzee genome (panTro5 chrY:15,306,523–15,356,698, with 100% span at 97.3% sequence identity). Alpha satellite and adjacent repeat in the chimpanzee genome that share limited sequence homology with human were determined used UCSC repeat table browser annotation^40^.

The location of the centromere across primate Y-chromosomes was determined by fluorescence *in situ* hybridization (FISH) (Supplementary Fig. 14). Preparation of mitotic chromosomes and BAC-based probes were carried according to standard procedures^41^. Primate cell lines were obtained from Coriell: *Pan paniscus* (Bonobo) AG05253; *Pan troglodytes* (Common Chimpanzee) S006006E. Male gorilla fibroblast cells were provided by Stephen O'Brien (National Cancer Institute, Frederick, MD) as previously discussed^42^. The HuRef cell line^34^ (GM25430) was provided through collaboration with Samuel Levy. BAC DNAs were isolated from bacteria stabs obtained from CHORI BACPAC. Metaphase spreads were obtained after a 1 h 15 min colcemid/karyomax (Gibco) treatment followed by incubation in a hypotonic solution. Cells were counterstained with 4′,6-diamidino-2-phenylindole (DAPI) (Vector). BAC DNA probes were labeled using Alexa flour dyes (488, green and 594, red) (ThermoFisher). The BAC probes were labeled with biotin 14-dATP by nick translation (Gibco). And the chromosomes were counterstained with DAPI. Microscopy, image acquisition, and processing were performed using standard procedures.

### Epigenetic mapping of centromere proteins.

To evaluate similarity between the HuRef DYZ3 reference model (GenBank: GJ212193) and our RP11 BAC-assembly we determined the relative frequency of each k-mer in the array (where *k* = 21, with a 1-bp slide taking into account both forward and reverse sequence orientation using Jellyfish software) normalized by the total number of observed k-mers (Supplementary Fig. 15), with the Pearson correlation coefficient. Enrichment across the RP11 Y assembly was determined using the log-transformed relative enrichment of each 50-mer frequency relative to the frequency of that 50-mer in background control (GEO Accession: GSE45497 ID: 200045497), as previously described^5^. If a 50-mer is not observed in the ChIP background the relative frequency was determined relative to the HuRef Sanger WGS read data (AADD00000000 WGSA)^34^. Average enrichment values were calculated for windows size 6 kb (Fig. 2). Additionally, CENP-A and C paired read data sets (GEO Accession: GSE60951 ID: 200060951)^43^ were merged (PEAR^27^, standard parameters) and mapped to all alpha satellite reference models in GRCh38. Reads that mapped specifically to the DYZ3 reference model were selected to study enrichment to the HOR array. The total number of bases mapped from CENP-A and CENP-C data versus the input controls was used to determine relative enrichment. Second, reads that mapped specifically to the DYZ3 reference model were aligned to the DYZ3 5.7 kb in consensus (indexed in tandem to avoid edge-effects), and read depth profiles were determined. To characterize enrichment outside of the DYZ3 array CENP-A, CENP-C and Input data were mapped directly to the RP11 Y-assembly. Reads mapping to the DYZ3 array were ignored. Read alignments were only considered outside of the DYZ3 array if no mismatches, insertions, or deletions were observed to the reference and if the read could be aligned to a single location (removing any reads with mapping score of 0). Sequence depth profiles were calculated by counting the number of bases at any position and normalizing by the total number of bases in each respective data set. Relative enrichment was obtained by taking the log-transformed normalized ratio of centromere protein (A or C) to Input.

### Statistics.

The Pearson correlation coefficient was used to determine a positive linear relationship in our data sets (as shown in Supplementary Figs. 10a and 15a). Simulation experiments using Illumina short read data were performed using 100 replicates. Representative gel image shown (Supplementary Fig. 6) was repeated ten times, or once for each BAC in our study, with consistent results. Representative Southern Blot (shown in Supplementary Fig. 4a), was repeated twice with different restriction enzymes with the same results. Centromere Y position analysis using FISH on a panel of primates were repeated at least two times, and results were invariable between experiments and between hybridization patterns within multiple metaphase spreads within a given experiment.

### Code availability.

This study used previously published software: alignments were performed using BLASR^23^ (version 1.3.1.124201) and BWA MEM^28^ (0.7.12-r1044). Consensus alignments were obtained using kalign^24^ (version 2.04). Global alignments of HORs used needle^44^ (EMBOSS:6.5.7.0). Repeat characterization was performed using RepeatMasker (Smit, AFA, Hubley, R & Green, P. *RepeatMasker Open-4.0*. 2013-2015; http://www.repeatmasker.org). Satellite monomers were determined using profile hidden Markov model (HMMER3)^26^. Jellyfish (version 2.0.0)^25^ was used to characterize k-mers. Illumina read simulations was performed using ART (version 2.5.8)^29^. PEAR^27^ (version 0.9.0) was used to merge paired read data. Comparative genomic analysis between human and chimpanzee were performed using UCSC Genome Browser liftOver^39^. Additional scripts used in preparing sequences before consensus generation are deposited in GitHub: https://github.com/khmiga/CENY.

### Life Sciences Reporting Summary.

Further information on experimental design is available in the Life Sciences Reporting Summary.

### Data availability.

Sequence data that support the findings of this study have been deposited in GenBank with reference to BioProject ID PRJNA397218, and SRA accession codes SRR5902337 and SRR5902355. BAC consensus sequences and RP11-CENY array assembly are deposited under GenBank accession numbers MF741337–MF741347.

## Additional information

**Publisher's note:** Springer Nature remains neutral with regard to jurisdictional claims in published maps and institutional affiliations.

## Supplementary Information

### Integrated supplementary information

Supplementary Figure 1 A brief overview of employed strategy. BAC-based assembly across the DYZ3 locus requires overlap among a few informative sequence variants, thus placing great importance on the accuracy of base-calls. Therefore, we employed the following strategy (outlined in flow chart and discussed in more detail below) to improve overall base quality. First, we derived a consensus from multiple alignments of 1D reads that span the full insert length for each BAC. Further, polishing steps were performed using re-alignment of all full-length nanopore reads for each BAC. As a result, each BAC sequencing project resulted in a single polished, BAC consensus sequence. To validate single copy variants, useful in an overlap-layout-assembly strategy, we included Illumina datasets for each BAC. Illumina data was not used to correct or validate variants observed multiple times within a given BAC sequence due to the reduced mapping quality

Supplementary Figure 2 MinION yield versus read length. Each subpanel corresponds to the yield in megabases vs read length for a particular BAC with the selected sequence used to generate the consensus sequence highlighted in grey and blue dotted lines providing information for the median value, or expected size of the full-length BAC.

Supplementary Figure 3 Distribution of array lengths estimates. Distribution of array lengths estimates for 96 individuals assigned to the R1b Y-haplogroups from the Phase 1 1000 genome project. The assembled DYZ3 array length for the RP11 donor genome is shown as a dashed blue line.

Supplementary Figure 4 DYZ3 array length estimates by pulse field gel electrophoresis (PFGE) Southern DYZ3 array length estimates by pulse field gel electrophoresis (PFGE) Southern using digests with a Y-haplogroup R1b matched individual (HuRef cell line). DNA digest is shown in top panel for 6 enzymes used with corresponding CHEF gel (providing data from one experiment), where flanking restriction sites on p-arm and q-arm are shown as corresponding bars with the spanning array (that lacks the presented restriction enzyme site) is shown as think black bar. Lane 7 is used as a negative control (GM12708 female cell line, available from Coriell Institute). Size estimates were made using chromosomes from *S. cerevisiae* strain YNN295 and lambda DNA as markers (marker sizes in megabase pairs at left). Size estimates assuming the RP11 DYZ3 assembly are presented in the table relative to the relative positions of restriction sites in the human reference assembly flanking the centromeric region (GRCh38). Representative Southern blot was repeated twice (data not shown), each time used a different panel of restriction enzymes and provided consistent results.

Supplementary Figure 5 CENY haplotype groupings Four frequent variants in the DYZ3 HOR unit (5875 bp) were used to identify 9 haplotype groupings. The DYZ3 consensus 5.8kb repeat is shown as a grey arrow, with the directionality of the repeat indicated. The position and DYZ3 consensus base are indicated on the bottom in white circles, black circles denote single base changes at that site that are frequent in the array. Color banding represents the variant patterns that defined the nine DYZ3 haplogroups. The entire 52 HOR array is shown on top with DYZ3 HOR haplotype patterns labeled. Nucleotide variant information associated with this figure is available in Supplementary Table 2.

Supplementary Figure 6 Titration of transposase to cut BACs once used in Longboard 1D Protocol Representative gel image from a pulsed-field gel electrophoresis assay to test NotIHF digest of BACs, and to assess titration of DNA:ONT transposase (‘dead’ FRM + ‘live’ FRM). Data shown for two BACs: RP11-482A22 (∼175 kb Control BAC from Xq24) and RP11-718M18 (∼209 kb DYZ3-containing BAC). Circularized BACs are indicated in purple. High fidelity NotI (NotI-HF; NEB R3189S) was used to identify insert sequence (blue) and vector sequence (orange). Addition to transposase (‘dead’ FRM + ‘live’ FRM) indicates that the the majority of linearized DNAs (light orange, transpoase-cut BAC) are full-length or only cut once. Representative gel image shown was repeated ten times, or once for each BAC in our study, with consistent results.

Supplementary Figure 7 Read length distribution of a standard BAC sequencing run. Read length distribution of a standard BAC sequencing run using Longboard MinION protocol. Shown above for BAC RP11-648J18, read number versus read length plots are converted to yield plots (i.e. Mb versus Read length). This is performed to identify BAC length min-max selection thresholds.

Supplementary Figure 8 Read sampling improves consensus. Consensus percent identity is provided for BAC vector sequence (alignments to Genbank:), for RP11-718M18 nanopore data. Full-length nanopore reads were sampled (10 reads, 30 reads, 60 reads, or 90 reads), and a representative consensus sequence was generated using kalign software. The BAC vector sequence was identified using HMMER trained on the pBACe3.6 sequence (Genbank: U80929.2), and percent identity was determined pairwise alignments (EMBOSS needle). We found that sampled 60 reads provided the best alignment score (97.99%), and that increasing the number of reads did not improve the final consensus sequence quality. Further, we found that taking 10 consensus sequences, derived from sampling 60 nanopore reads, provided the best overall consensus value, with an ∼1% improvement.

Supplementary Figure 9 Comparisons of 5-mer enrichment and biases. As shown for the RP11-718M18 data, (a) Illumina 5-mer frequencies relative to 5-mer frequencies for a synthetic BAC insert assuming no variants, (b) Illumina 5-mer frequency relative to the reads obtained from the MinION (Albacore base-calling), (c) Illumina 5-mer frequency relative to consensus polished RP11-718M18 nanopore data, (d) investigating the expected proportion of homopolymers (AAAA/TTTT), we observe a correction in calls in our polishing step, which agrees with data from the Illumina read database.

Supplementary Figure 10 Illumina read depth to estimate copy number. Illumina read depth to estimate copy number. (a) Illumina BAC resequencing data are concordant for DYZ3 repeat copy estimates (r=0.9694; Pearson's correlation of corresponding Illumina and nanopore data for each of the 10 BACs). Using k-mer (where k=21) counts for sites specific to the vector sequence we can determine a range of expected depth or frequency to identify single copy sites for each BAC sequence library, as shown for RP-11 718M18 (b) Overlapping 21-mers with frequency counts within the range of the vector sequence for each BAC library is useful in identifying informative satellite variants (c).

Supplementary Figure 11 Illumina read coverage necessary for sequence identity improvement. To test the illumine sequence coverage necessary for our consensus polishing strategy we simulated a range (20x-100x, as shown on the x-axis) of Illumina paired read datasets for a known 73kb control region on chrYp that is spanned by our RP11-531P03 BAC. After filtering out reads with low mapping scores (>= mapQ score of 20), we evaluated the resulting consensus sequence identity relative to the known assembled region on the Y chromosome (hg38 chrY:10137141-10210167), as shown as a frequency of correct called bases, or matches with the control region over the total length of the hg38 reference sequence. Illumina support for a given base call is determined by sequence coverage and the number of mapped reads (data shown for the frequency range of 0.6 to 0.9). The sequence identity observed using the entire Illumina paired read data set is shown as a grey dotted line.

Supplementary Figure 12 Investigating our mapping strategy using a simulated DYZ3 reference array. We introduced a single sequence base-change(A, T, C,or G) at random into a simulated DYZ3 array (otherwise containing 10X exact copies of the HOR repeat). Illumina paired reads were simulated either from the DYZ3 array before introducing the variant and after introducing the variant. Paired reads, either containing the variant sequence or not, were mapped back to the simulated reference array containing the variant. We performed this simulation altering both coverage (20-100x) and Illumina substitution error rate. We reported the average read coverage supporting the introduced reference variant. We found consistent high coverage support for the experiments where the reads contained the variant base (blue), which was not dependent on sequence coverage but was affected by Illumina error rate. Conversely, we found little read alignment support (grey) for Illumina datasets that did not contain the variant.

Supplementary Figure 13 Evidence for satellite variants in overlap region between repeats 28-35. Informative variants useful in ensuring proper overlap are shown for repeat 30 (yellow) and repeat 35 (light purple). Support for five variant positions are shown for all BACs (blue) with the reference base indicated in red. Relevant alignments for each variant are provided with shared variant bases/positions indicated in red.

Supplementary Figure 14 The Y centromere location is not shared among the great apes. (a) FISH images of DAPI stained chromosomes with BAC probes CH251 104M09 (red) and 656P08 (green), demonstrate the relative position of DNAs that are known to flank the human Y centromeric region (∼5.5 Mb apart). The same probes are known to lie adjacent to one another in the chimpanzee assembled Y chromosome 202 kb apart and do not span the chimpanzee centromere (as indicated in FISH image where centromere primary constrictions are highlighted with a white arrow). In chimpanzee (*Pan troglodytes*, PTR), bonobo (*Pan paniscus*, PPA), gorilla (*Gorilla gorilla*, GGO) these probes lie close to one another and are not observed to span the centromere primary constriction as in human (*Homo sapiens*, HSA). FISH experiments were repeated at least two times, and results were invariable between experiments and between hybridization patterns within multiple metaphase spreads within any given experiment. (b) Although centromere position is not shared, a small region of divergent alpha satellite from the Y p-Arm is observed to share high identity (97% over 11.9 kb, including junction with a shared L1PA3 insertion) in the chimpanzee assembled genome in the syntenic position. Additional alpha satellite is present on the chimpanzee assembly in this position, yet it has little to no sequence conservation with human (∼38.4kb with <70% sequence identity in pairwise comparisons of full length monomers).

Supplementary Figure 15 Epigenetic characterization of the Y Centromere Genomic characterization of the Y centromere is determined epigenetically by the enrichment of inner kinetochore proteins known to be critical for centromere identity and kinetochore assembly (Centromere protein A and C, CENP-A and CENP-C) ^4–6^. Using R1-b Y-haplogroup matched individual (HuRef ^7^) DYZ3 reference model array in GRCh38 ^8^ we demonstrate by k-mer comparing relative k-mer frequencies that the sequences are largely concordant (a) (r=0.9622 Pearson correlation of 13,344 50-mers shared between RP11-CENY and the reference model, GJ212193.1). Mapping publically available donor-matched CENP-A and CENP-C ChIP-Seq data ^9^ we determine high relative enrichment of the DYZ3 HOR sequence (b). Merged paired reads of plotted base pair depth provides evidence for nucleosome phasing along the length of the 5785 bp DYZ3 consensus repeat for CENP-A (red) and for the array-bound protein CENP-C (blue), and shown for technical replicates in grey (c,d). By eliminating multi-mapping reads, we identified enrichment directly adjacent to the DYZ3 array (as shown averaged for each 100 bp window in panel e), where enrichment is observed across the HOR transition zone and into the divergent alpha satellite monomers.

### Supplementary information

Supplementary Text and Figures Supplementary Figures 1–15 (PDF 2181 kb)

Life Sciences Reporting Summary (PDF 181 kb)

Supplementary Table 1 Throughput for each of the BAC runs. (PDF 149 kb)

Supplementary Dataset 1 Illumina validated variant sequence information. Variant summary information is provided for each BAC after Illumina validation. Variant positions are characterized as a mismatch, insertion (BAC introduced base not observed in DYZ3 repeat), deletion (in the BAC sequence relative to the DYZ3 repeat). In addition to the 7 structural HOR variants (6.0kb) distributed in the array, 9 single nucleotide variants were used to confirm the order of the DYZ3-containing BACs. Single copy variants (i.e. substitution (SUB), deletion (DEL), or insertion (INS)) were identified for each BAC. The variant was considered validated if observed in the Illumina data (by 21-mer and/or Illumina Q20 alignment data). Further, the variant must be observed across all BACs in the corresponding position, which also have Illumina support. (XLSX 66 kb)

Supplementary Dataset 2 CENP-A enrichment of RP11-CENY 50-mers. CENP-A ChIP-seq enrichment values are provided for each 50-mer in the RP11-CENY assembly. Column 1 provides the 50-mer fasta sequence, column 2 provides the CENP-A read count, column 3 is the normalized read depth frequency relative to the total read count in the previously published CENP-A ChIP Seq dataset (GEO Sample ID GSM1105684), column 4 provides the control/background read count, column 5 is the normalized read depth frequency relative to the total read count in the previously published Background ChIP Seq dataset (GEO Sample ID GSM1105685), and column 6 is the relative enrichment score. (TXT 1495 kb)

Supplementary Dataset 3 CENP-A enrichment of RP11-CENY peaks. A bedfile of CENP-A ChIP-seq enrichment values for each 100 bp non-overlapping window of the RP11-CENY assembly. Column 1 provides the assembly name, column 2 provides the start position, column 3 is the end position, column 4 is the average enrichment value. (TXT 130 kb)

## Data Availability

BioProject
PRJNA397218 GenBank/EMBL/DDBJ
MF741337
MF741347 Sequence Read Archive
SRR5902337
SRR5902355 GenBank/EMBL/DDBJ
U80929.2 Gene Expression Omnibus
200045497
200060951
GSE45497
GSE60951
