# Supplementary figures and images for: Linear assembly of a human centromere on the Y chromosome
Source: Nat Biotechnol. 2018 Mar 19;36(4):321–3. doi: 10.1038/nbt.4109 (PMC5886786; doi:10.1038/nbt.4109)

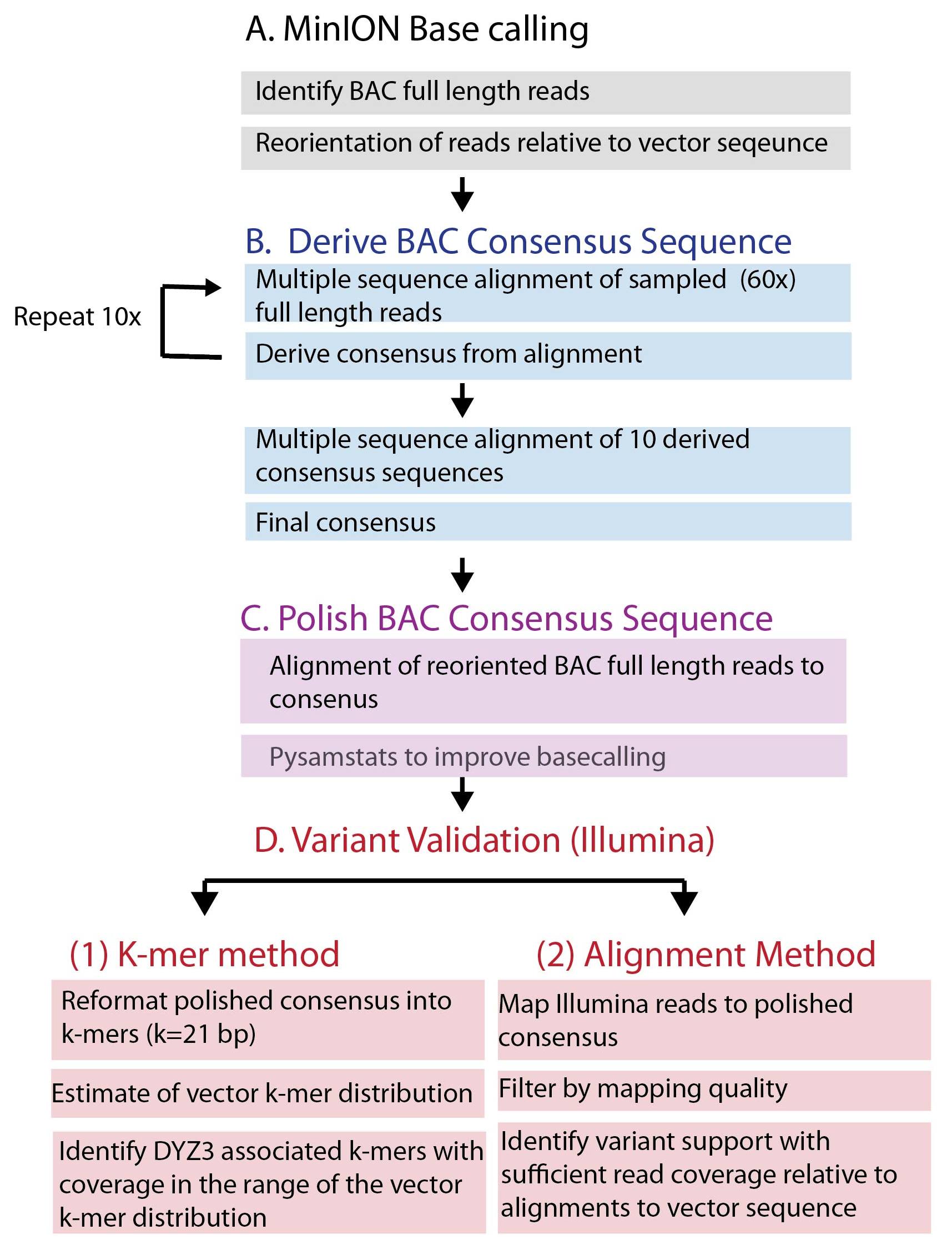

Supplement: A brief overview of employed strategy. — BAC-based assembly across the DYZ3 locus requires overlap among a few informative sequence variants, thus placing great importance on the accuracy of base-calls. Therefore, we employed the following strategy (outlined in flow chart and discussed in more detail below) to improve overall base quality. First, we derived a consensus from multiple alignments of 1D reads that span the full insert length for each BAC. Further, polishing steps were performed using re-alignment of all full-length nanopore reads for each BAC. As a result, each BAC sequencing project resulted in a single polished, BAC consensus sequence. To validate single copy variants, useful in an overlap-layout-assembly strategy, we included Illumina datasets for each BAC. Illumina data was not used to correct or validate variants observed multiple times within a given BAC sequence due to the reduced mapping quality [file 41587_2018_Article_BFnbt4109_Fig3_ESM.jpg]

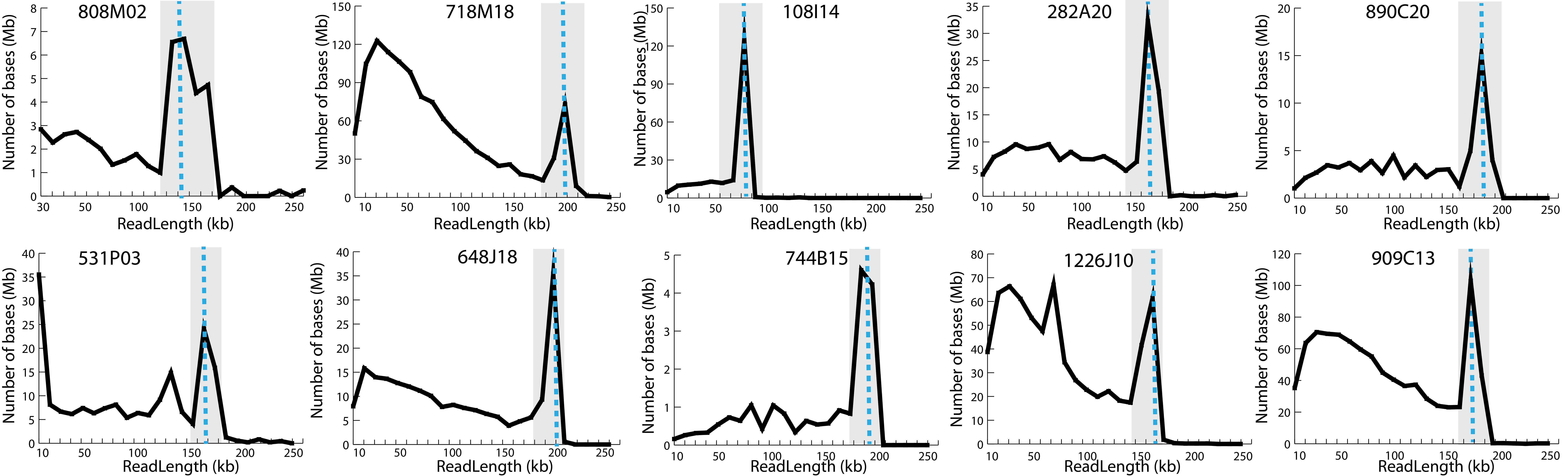

Supplement: MinION yield versus read length. — Each subpanel corresponds to the yield in megabases vs read length for a particular BAC with the selected sequence used to generate the consensus sequence highlighted in grey and blue dotted lines providing information for the median value, or expected size of the full-length BAC. [file 41587_2018_Article_BFnbt4109_Fig4_ESM.jpg]

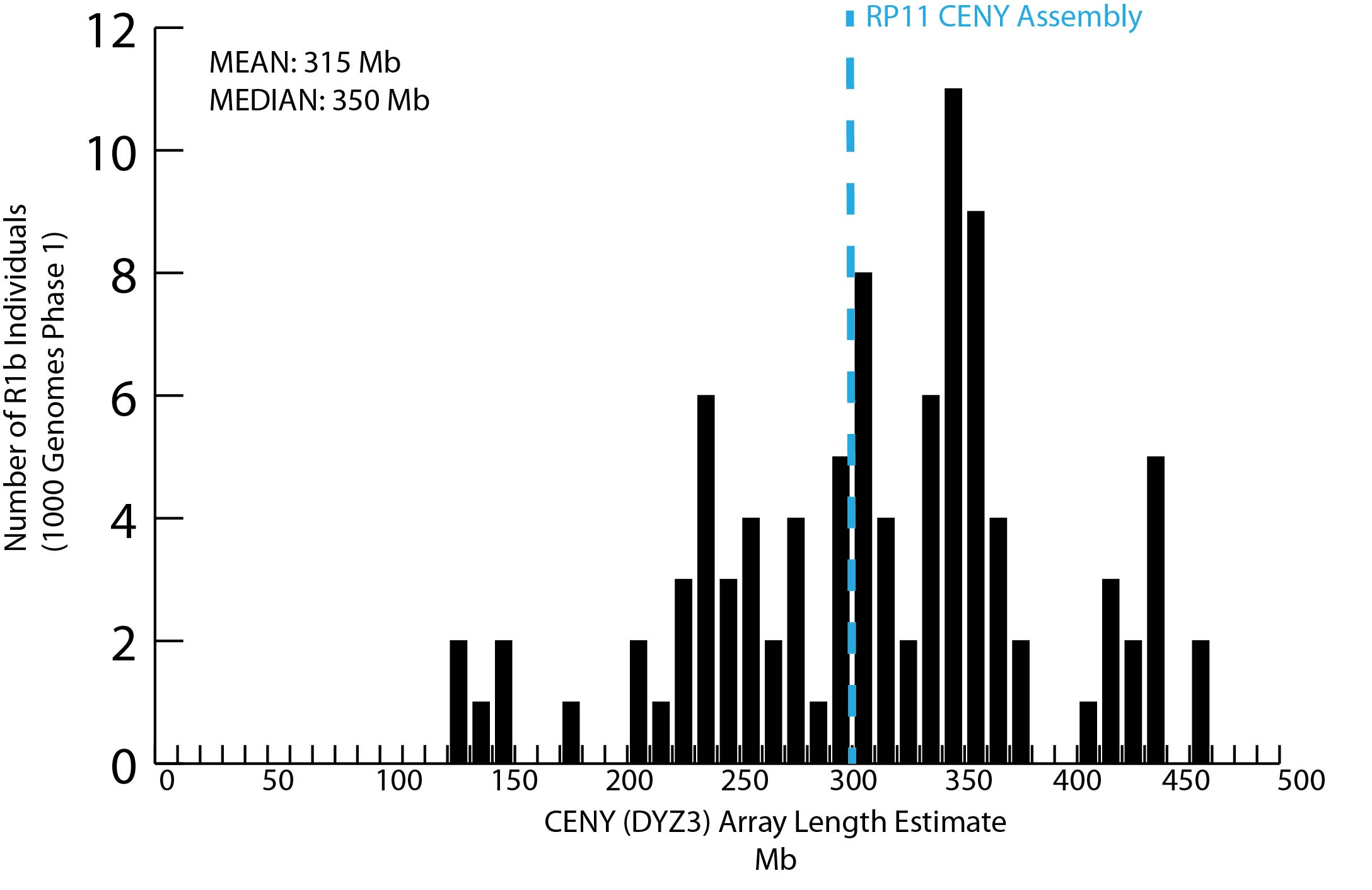

Supplement: Distribution of array lengths estimates. — Distribution of array lengths estimates for 96 individuals assigned to the R1b Y-haplogroups from the Phase 1 1000 genome project. The assembled DYZ3 array length for the RP11 donor genome is shown as a dashed blue line. [file 41587_2018_Article_BFnbt4109_Fig5_ESM.jpg]

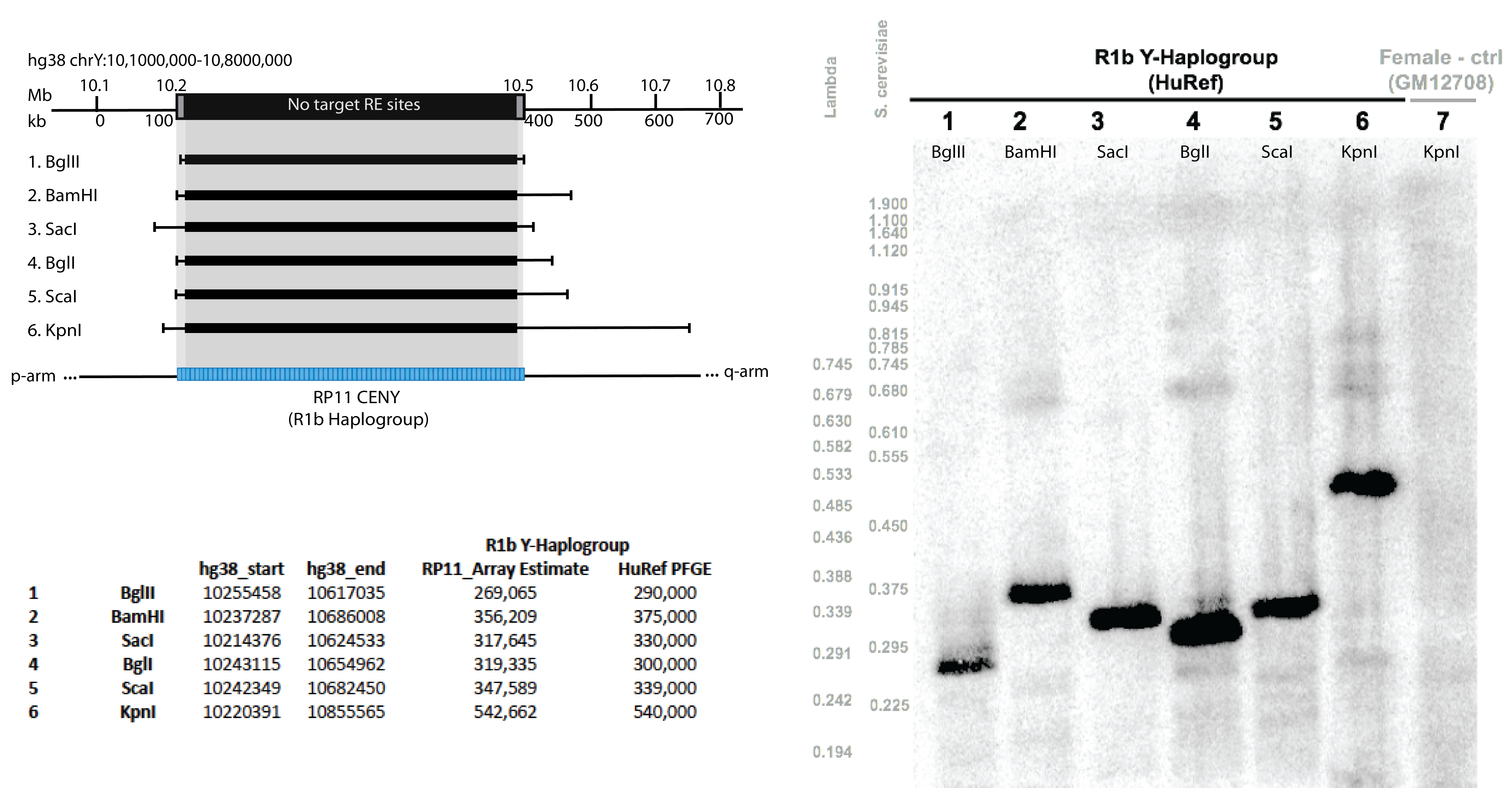

Supplement: DYZ3 array length estimates by pulse field gel electrophoresis (PFGE) Southern — using digests with a Y-haplogroup R1b matched individual (HuRef cell line). DNA digest is shown in top panel for 6 enzymes used with corresponding CHEF gel (providing data from one experiment), where flanking restriction sites on p-arm and q-arm are shown as corresponding bars with the spanning array (that lacks the presented restriction enzyme site) is shown as think black bar. Lane 7 is used as a negative control (GM12708 female cell line, available from Coriell Institute). Size estimates were made using chromosomes from S. cerevisiae strain YNN295 and lambda DNA as markers (marker sizes in megabase pairs at left). Size estimates assuming the RP11 DYZ3 assembly are presented in the table relative to the relative positions of restriction sites in the human reference assembly flanking the centromeric region (GRCh38). Representative Southern blot was repeated twice (data not shown), each time used a different panel of restriction enzymes and provided consistent results. [file 41587_2018_Article_BFnbt4109_Fig6_ESM.jpg]

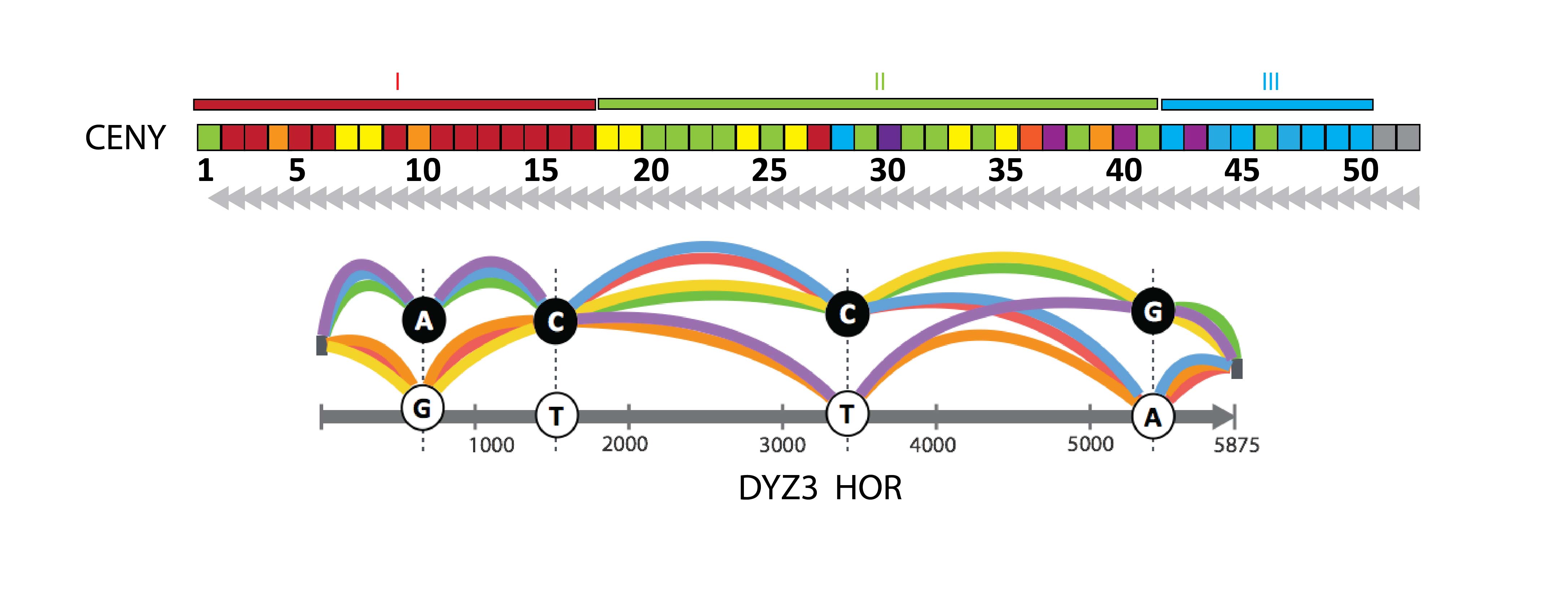

Supplement: CENY haplotype groupings — Four frequent variants in the DYZ3 HOR unit (5875 bp) were used to identify 9 haplotype groupings. The DYZ3 consensus 5.8kb repeat is shown as a grey arrow, with the directionality of the repeat indicated. The position and DYZ3 consensus base are indicated on the bottom in white circles, black circles denote single base changes at that site that are frequent in the array. Color banding represents the variant patterns that defined the nine DYZ3 haplogroups. The entire 52 HOR array is shown on top with DYZ3 HOR haplotype patterns labeled. Nucleotide variant information associated with this figure is available in Supplementary Table 2. [file 41587_2018_Article_BFnbt4109_Fig7_ESM.jpg]

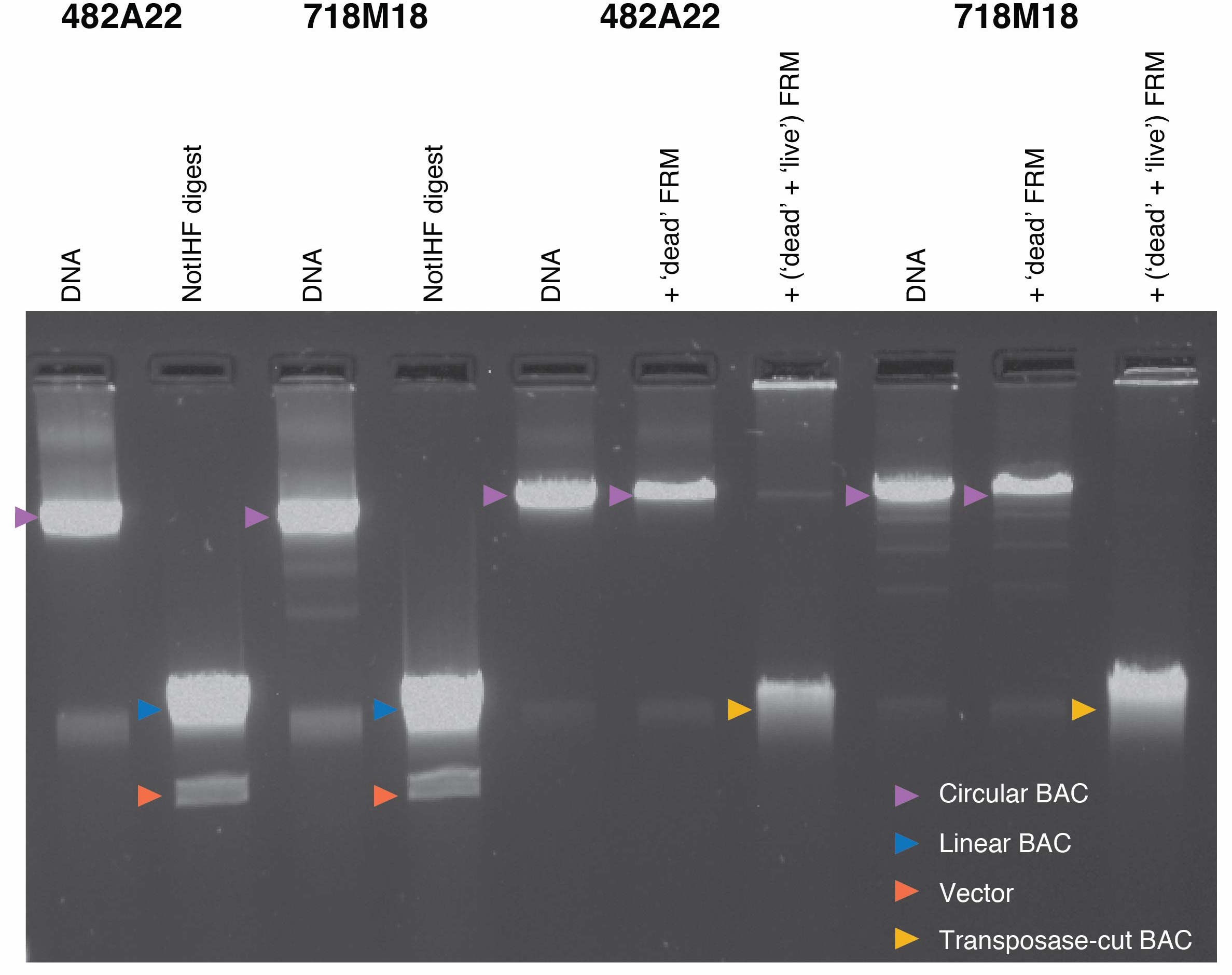

Supplement: Titration of transposase to cut BACs once used in Longboard 1D Protocol — Representative gel image from a pulsed-field gel electrophoresis assay to test NotIHF digest of BACs, and to assess titration of DNA:ONT transposase (‘dead’ FRM + ‘live’ FRM). Data shown for two BACs: RP11-482A22 (∼175 kb Control BAC from Xq24) and RP11-718M18 (∼209 kb DYZ3-containing BAC). Circularized BACs are indicated in purple. High fidelity NotI (NotI-HF; NEB R3189S) was used to identify insert sequence (blue) and vector sequence (orange). Addition to transposase (‘dead’ FRM + ‘live’ FRM) indicates that the the majority of linearized DNAs (light orange, transpoase-cut BAC) are full-length or only cut once. Representative gel image shown was repeated ten times, or once for each BAC in our study, with consistent results. [file 41587_2018_Article_BFnbt4109_Fig8_ESM.jpg]

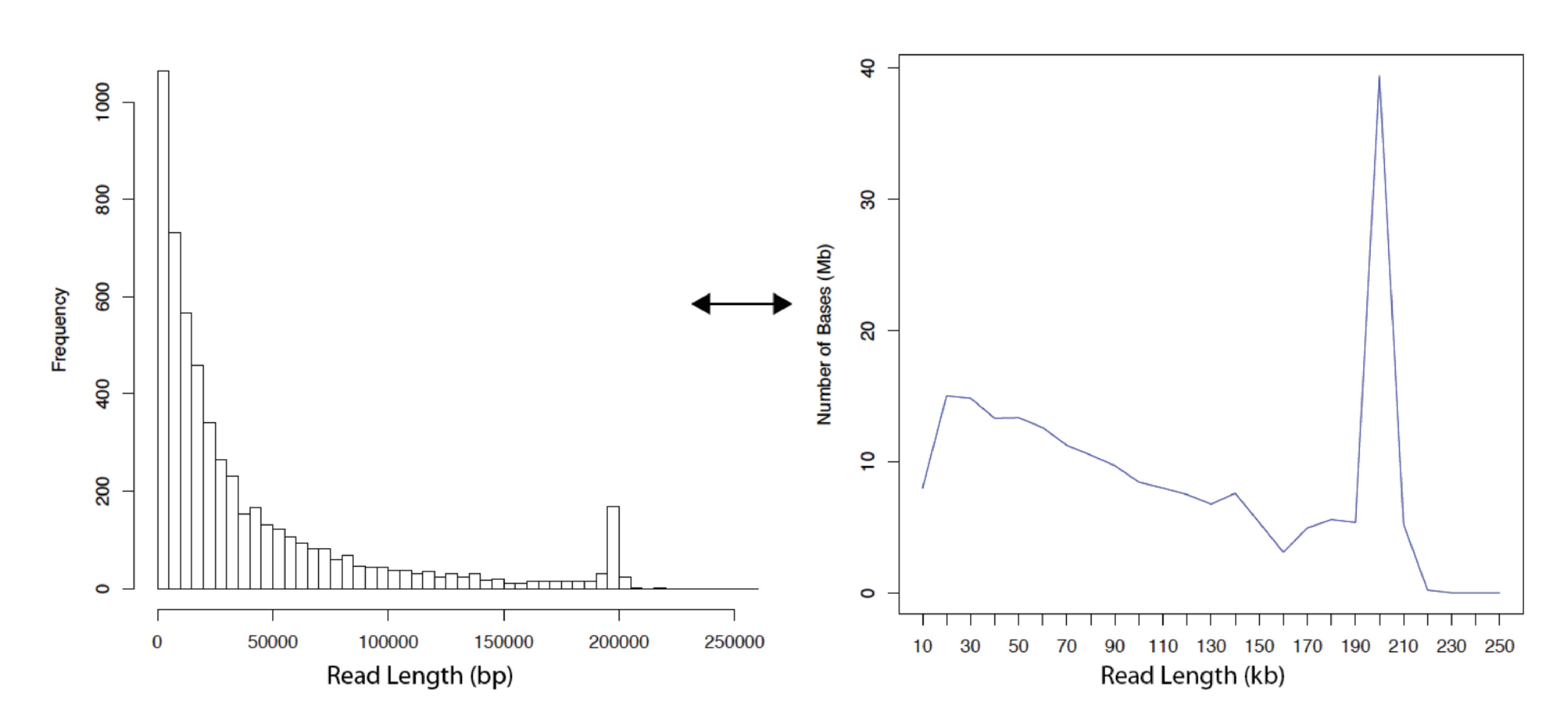

Supplement: Read length distribution of a standard BAC sequencing run. — Read length distribution of a standard BAC sequencing run using Longboard MinION protocol. Shown above for BAC RP11-648J18, read number versus read length plots are converted to yield plots (i.e. Mb versus Read length). This is performed to identify BAC length min-max selection thresholds. [file 41587_2018_Article_BFnbt4109_Fig9_ESM.jpg]

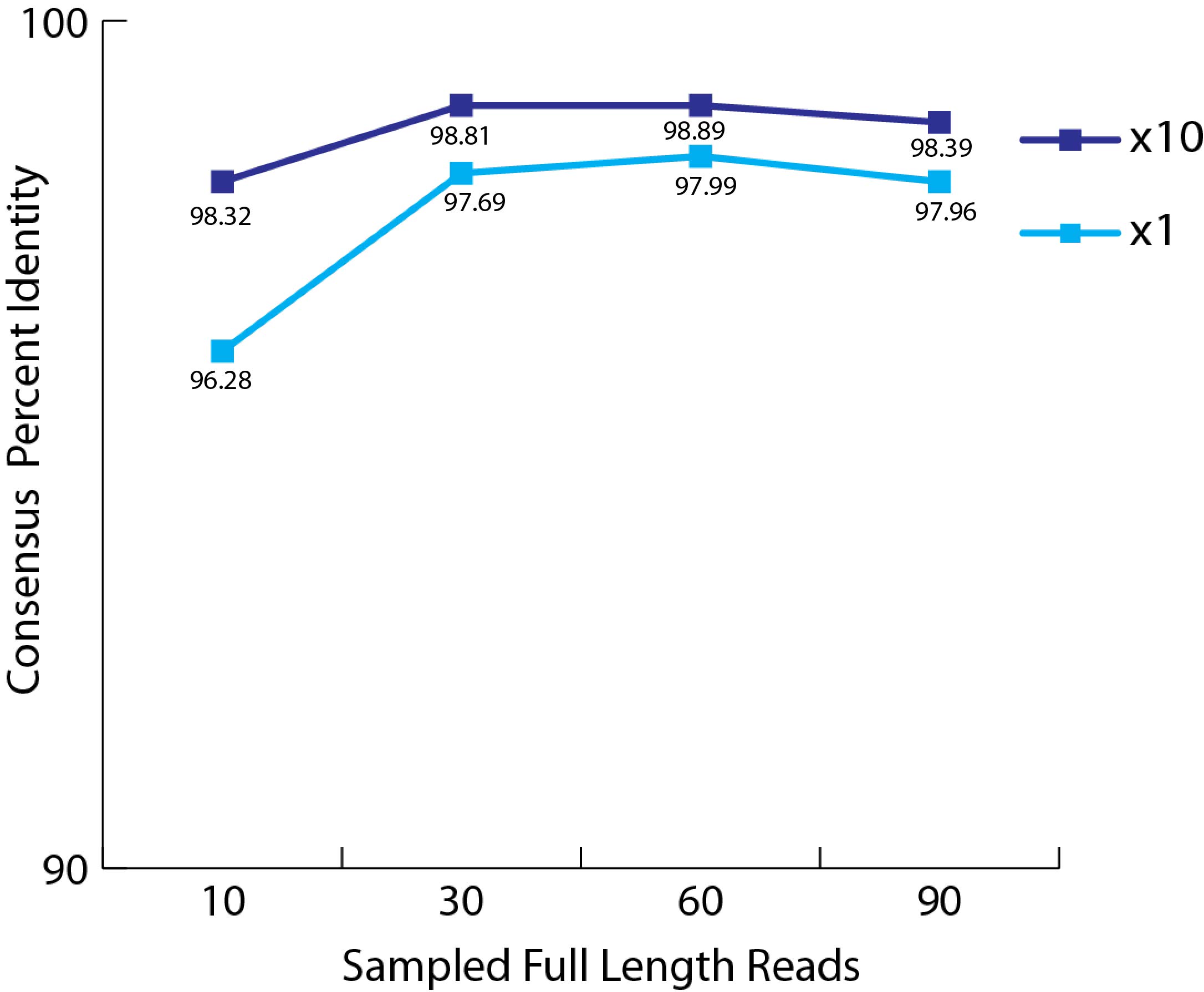

Supplement: Read sampling improves consensus. — Consensus percent identity is provided for BAC vector sequence (alignments to Genbank:), for RP11-718M18 nanopore data. Full-length nanopore reads were sampled (10 reads, 30 reads, 60 reads, or 90 reads), and a representative consensus sequence was generated using kalign software. The BAC vector sequence was identified using HMMER trained on the pBACe3.6 sequence (Genbank: U80929.2), and percent identity was determined pairwise alignments (EMBOSS needle). We found that sampled 60 reads provided the best alignment score (97.99%), and that increasing the number of reads did not improve the final consensus sequence quality. Further, we found that taking 10 consensus sequences, derived from sampling 60 nanopore reads, provided the best overall consensus value, with an ∼1% improvement. [file 41587_2018_Article_BFnbt4109_Fig10_ESM.jpg]

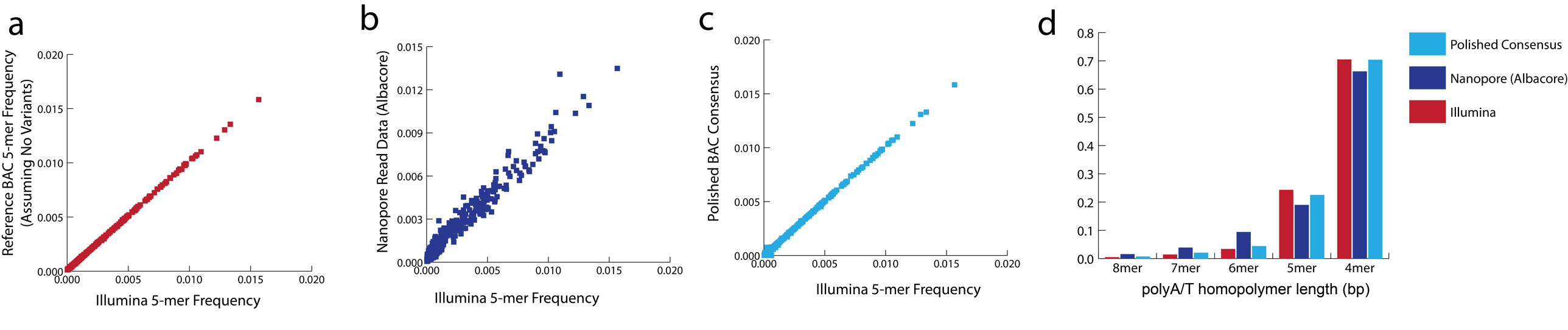

Supplement: Comparisons of 5-mer enrichment and biases. — As shown for the RP11-718M18 data, (a) Illumina 5-mer frequencies relative to 5-mer frequencies for a synthetic BAC insert assuming no variants, (b) Illumina 5-mer frequency relative to the reads obtained from the MinION (Albacore base-calling), (c) Illumina 5-mer frequency relative to consensus polished RP11-718M18 nanopore data, (d) investigating the expected proportion of homopolymers (AAAA/TTTT), we observe a correction in calls in our polishing step, which agrees with data from the Illumina read database. [file 41587_2018_Article_BFnbt4109_Fig11_ESM.jpg]

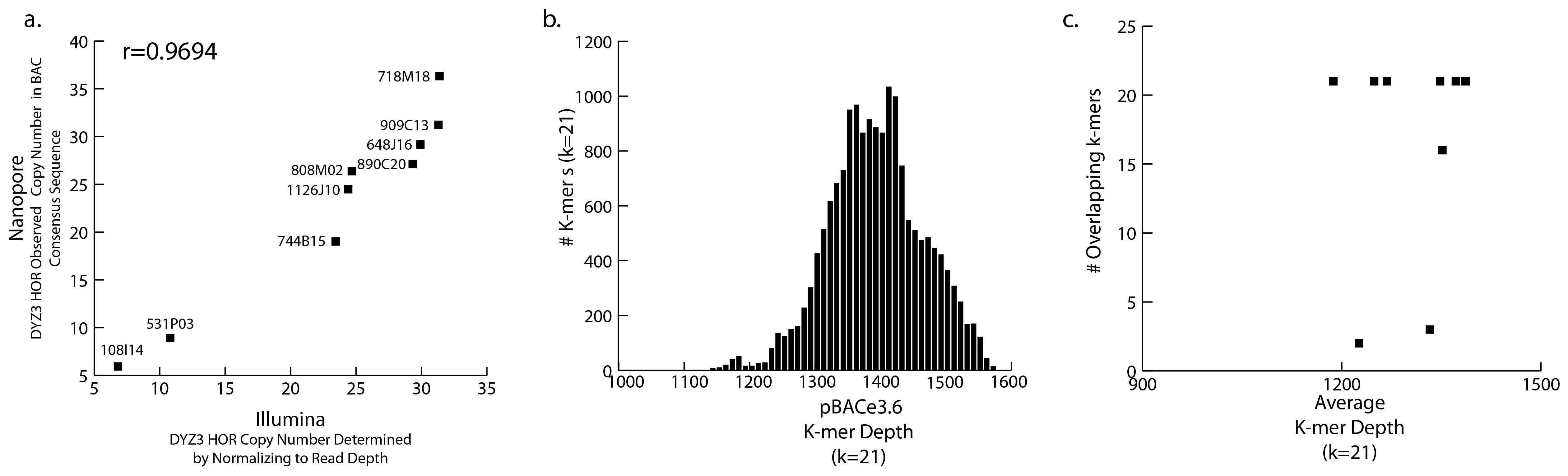

Supplement: Illumina read depth to estimate copy number. — (a) Illumina BAC resequencing data are concordant for DYZ3 repeat copy estimates (r=0.9694; Pearson's correlation of corresponding Illumina and nanopore data for each of the 10 BACs). Using k-mer (where k=21) counts for sites specific to the vector sequence we can determine a range of expected depth or frequency to identify single copy sites for each BAC sequence library, as shown for RP-11 718M18 (b) Overlapping 21-mers with frequency counts within the range of the vector sequence for each BAC library is useful in identifying informative satellite variants (c). [file 41587_2018_Article_BFnbt4109_Fig12_ESM.jpg]

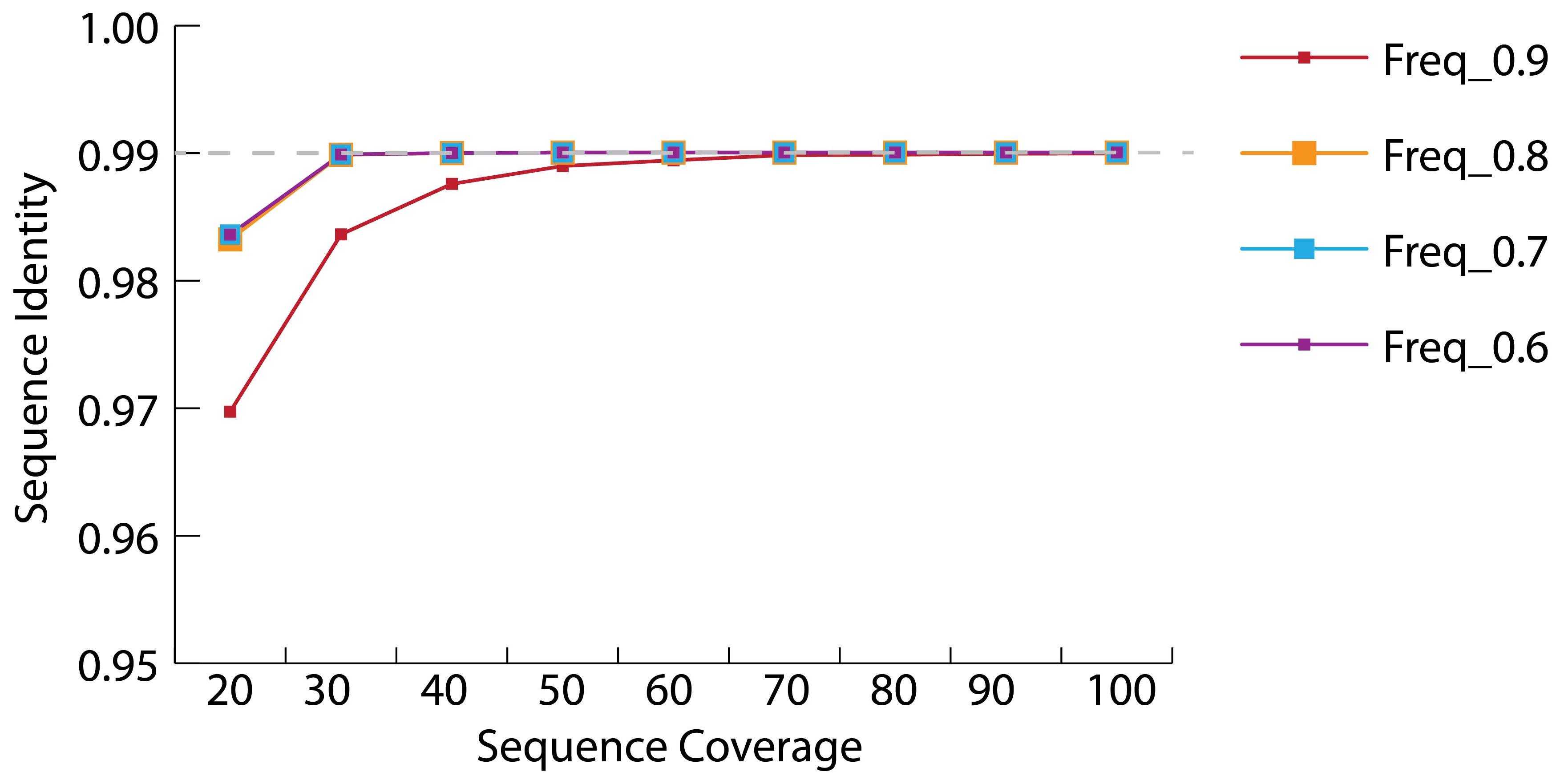

Supplement: Illumina read coverage necessary for sequence identity improvement. — To test the illumine sequence coverage necessary for our consensus polishing strategy we simulated a range (20x-100x, as shown on the x-axis) of Illumina paired read datasets for a known 73kb control region on chrYp that is spanned by our RP11-531P03 BAC. After filtering out reads with low mapping scores (>= mapQ score of 20), we evaluated the resulting consensus sequence identity relative to the known assembled region on the Y chromosome (hg38 chrY:10137141-10210167), as shown as a frequency of correct called bases, or matches with the control region over the total length of the hg38 reference sequence. Illumina support for a given base call is determined by sequence coverage and the number of mapped reads (data shown for the frequency range of 0.6 to 0.9). The sequence identity observed using the entire Illumina paired read data set is shown as a grey dotted line. [file 41587_2018_Article_BFnbt4109_Fig13_ESM.jpg]

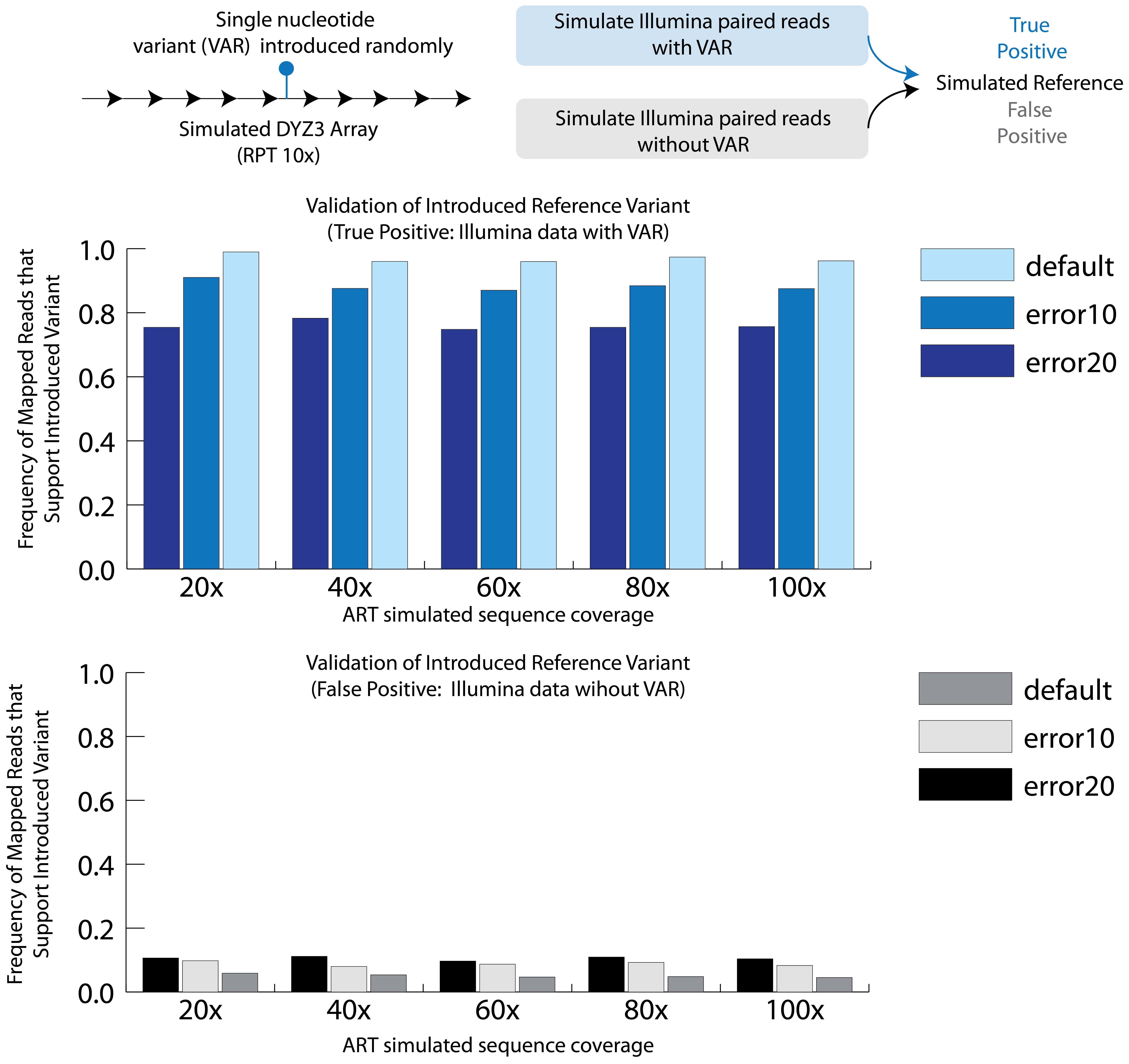

Supplement: Investigating our mapping strategy using a simulated DYZ3 reference array. — We introduced a single sequence base-change(A, T, C,or G) at random into a simulated DYZ3 array (otherwise containing 10X exact copies of the HOR repeat). Illumina paired reads were simulated either from the DYZ3 array before introducing the variant and after introducing the variant. Paired reads, either containing the variant sequence or not, were mapped back to the simulated reference array containing the variant. We performed this simulation altering both coverage (20-100x) and Illumina substitution error rate. We reported the average read coverage supporting the introduced reference variant. We found consistent high coverage support for the experiments where the reads contained the variant base (blue), which was not dependent on sequence coverage but was affected by Illumina error rate. Conversely, we found little read alignment support (grey) for Illumina datasets that did not contain the variant. [file 41587_2018_Article_BFnbt4109_Fig14_ESM.jpg]

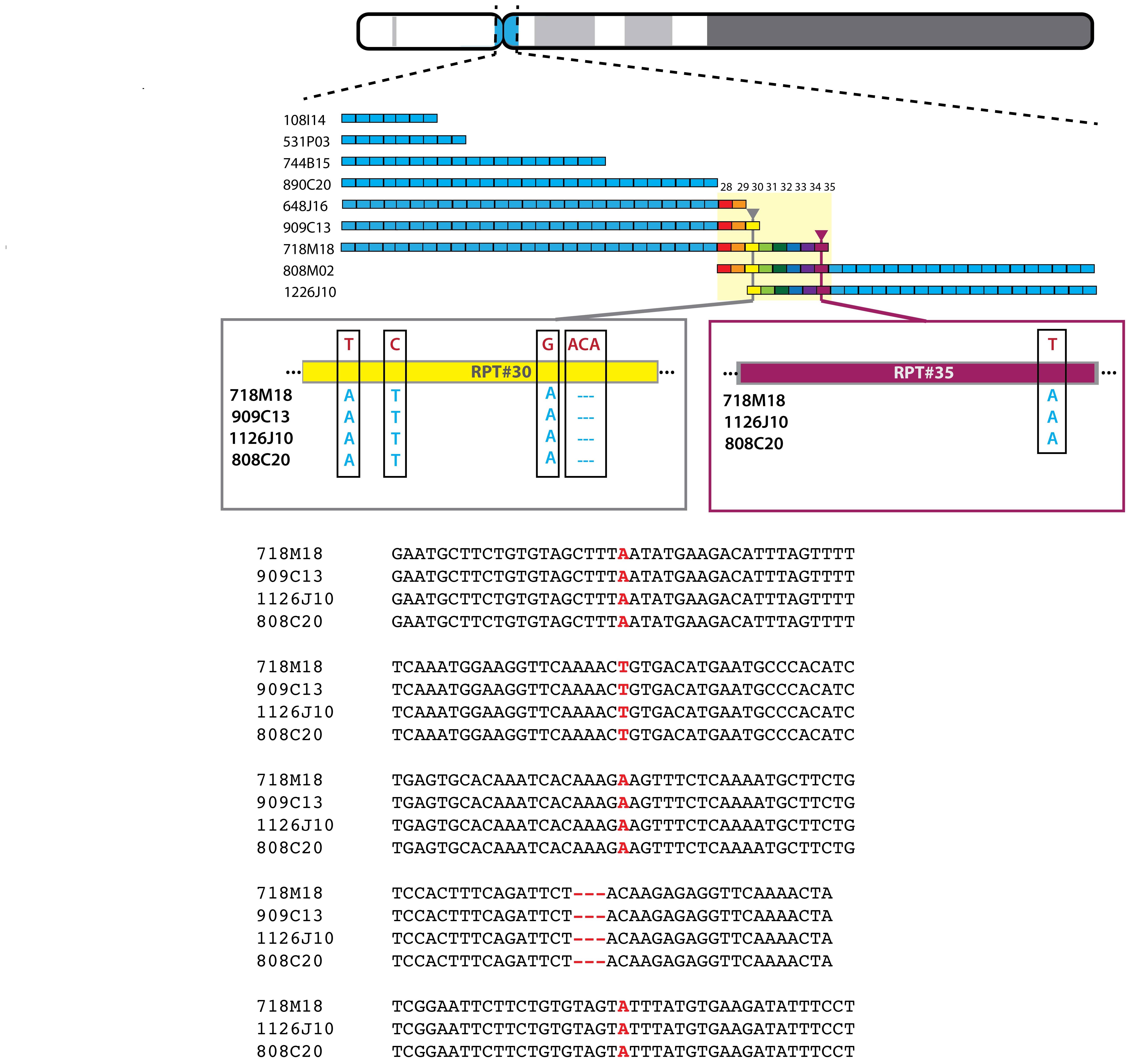

Supplement: Evidence for satellite variants in overlap region between repeats 28-35. — Informative variants useful in ensuring proper overlap are shown for repeat 30 (yellow) and repeat 35 (light purple). Support for five variant positions are shown for all BACs (blue) with the reference base indicated in red. Relevant alignments for each variant are provided with shared variant bases/positions indicated in red. [file 41587_2018_Article_BFnbt4109_Fig15_ESM.jpg]

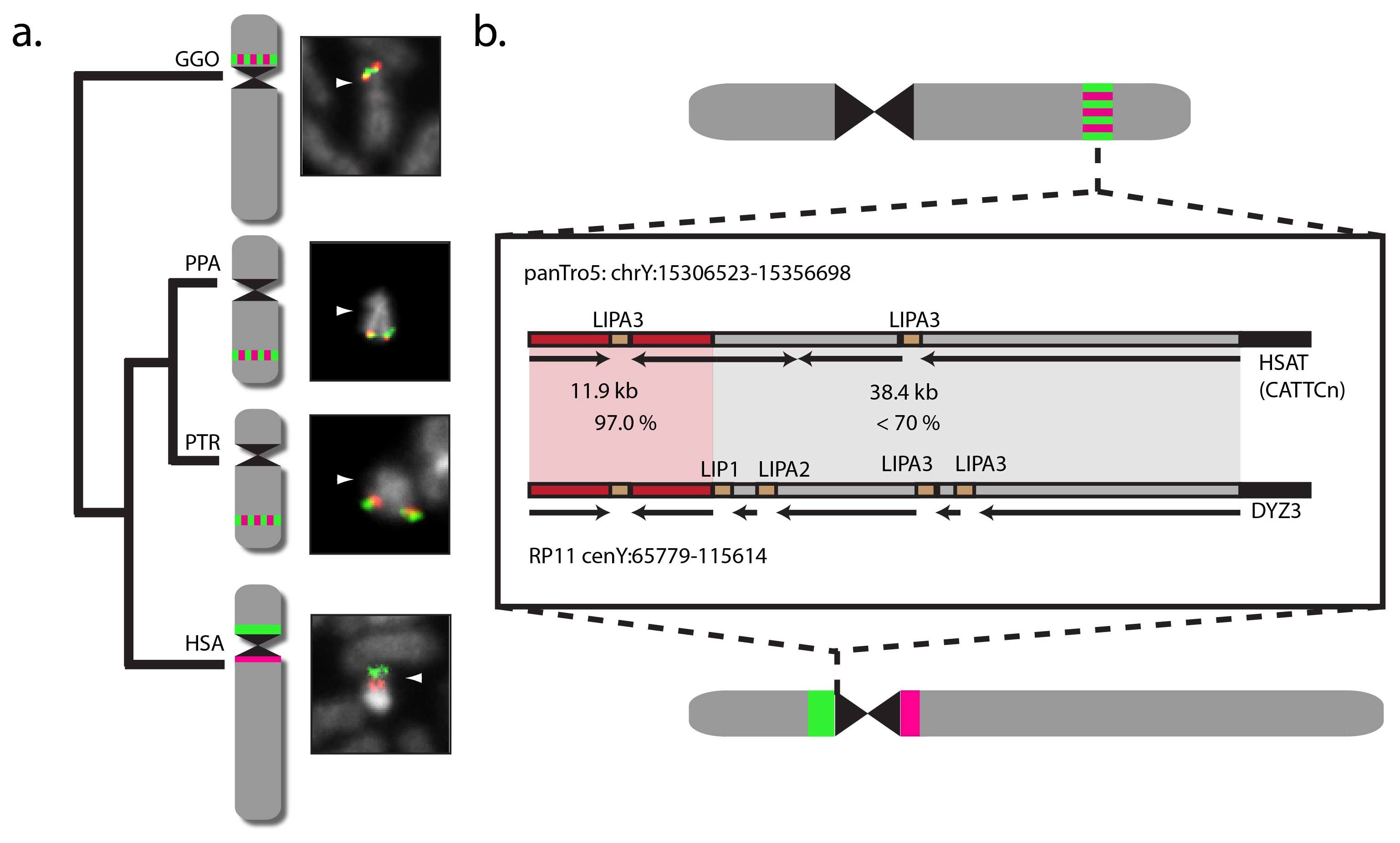

Supplement: The Y centromere location is not shared among the great apes. — (a) FISH images of DAPI stained chromosomes with BAC probes CH251 104M09 (red) and 656P08 (green), demonstrate the relative position of DNAs that are known to flank the human Y centromeric region (∼5.5 Mb apart). The same probes are known to lie adjacent to one another in the chimpanzee assembled Y chromosome 202 kb apart and do not span the chimpanzee centromere (as indicated in FISH image where centromere primary constrictions are highlighted with a white arrow). In chimpanzee (Pan troglodytes, PTR), bonobo (Pan paniscus, PPA), gorilla (Gorilla gorilla, GGO) these probes lie close to one another and are not observed to span the centromere primary constriction as in human (Homo sapiens, HSA). FISH experiments were repeated at least two times, and results were invariable between experiments and between hybridization patterns within multiple metaphase spreads within any given experiment. (b) Although centromere position is not shared, a small region of divergent alpha satellite from the Y p-Arm is observed to share high identity (97% over 11.9 kb, including junction with a shared L1PA3 insertion) in the chimpanzee assembled genome in the syntenic position. Additional alpha satellite is present on the chimpanzee assembly in this position, yet it has little to no sequence conservation with human (∼38.4kb with <70% sequence identity in pairwise comparisons of full length monomers). [file 41587_2018_Article_BFnbt4109_Fig16_ESM.jpg]
