# Supplementary material for: Linear assembly of a human centromere on the Y chromosome
Source: Nat Biotechnol. 2018 Mar 19;36(4):321–3. doi: 10.1038/nbt.4109 (PMC5886786; doi:10.1038/nbt.4109)
Supplement: Supplementary file 17 — Life Sciences Reporting Summary (PDF 181 kb) [file 41587_2018_BFnbt4109_MOESM23_ESM.pdf]

nature research

Corresponding author(s): Dr. Karen H. Miga

☐ Initial submission ☐ Revised version ☒ Final submission

Life Sciences Reporting Summary

Nature Research wishes to improve the reproducibility of the work that we publish. This form is intended for publication with all accepted life science papers and provides structure for consistency and transparency in reporting. Every life science submission will use this form; some list items might not apply to an individual manuscript, but all fields must be completed for clarity.

For further information on the points included in this form, see Reporting Life Sciences Research. For further information on Nature Research policies, including our data availability policy, see Authors & Referees and the Editorial Policy Checklist.

Experimental design

1. Sample size

Describe how sample size was determined.

A single sample (RP11) was sequenced and so this is not applicable.

2. Data exclusions

Describe any data exclusions.

We excluded short reads from our analysis - defined as reads that are not represent the full length of the BAC insert and/or offered less than 4 kb of vector sequence.

3. Replication

Describe whether the experimental findings were reliably reproduced.

We reliably reproduced the MinION base statistics and consensus polishing results using a control RP11-482A22 BAC from the X chromosome

4. Randomization

Describe how samples/organisms/participants were allocated into experimental groups.

Read ids were randomly selected from a scrambled index of all full length reads. We optimized our study by obtaining random sampling of 10, 30, 60, 90 reads. Improvements were 98-99% identity for 60x, with only slight improvements with greater read depth.

5. Blinding

Describe whether the investigators were blinded to group allocation during data collection and/or analysis.

No blinding was required for this study.

Note: all studies involving animals and/or human research participants must disclose whether blinding and randomization were used.

6. Statistical parameters

For all figures and tables that use statistical methods, confirm that the following items are present in relevant figure legends (or in the Methods section if additional space is needed).

n/a	Confirmed	
☐	☒	The exact sample size (n) for each experimental group/condition, given as a discrete number and unit of measurement (animals, litters, cultures, etc.)
☐	☒	A description of how samples were collected, noting whether measurements were taken from distinct samples or whether the same sample was measured repeatedly
☐	☒	A statement indicating how many times each experiment was replicated
☒	☐	The statistical test(s) used and whether they are one- or two-sided (note: only common tests should be described solely by name; more complex techniques should be described in the Methods section)
☒	☐	A description of any assumptions or corrections, such as an adjustment for multiple comparisons
☒	☐	The test results (e.g. P values) given as exact values whenever possible and with confidence intervals noted
☐	☒	A clear description of statistics including central tendency (e.g. median, mean) and variation (e.g. standard deviation, interquartile range)
☒	☐	Clearly defined error bars

See the web collection on statistics for biologists for further resources and guidance.

Nature Biotechnology: doi:10.1038/nbt.4109

Software

Policy information about availability of computer code

7. Software

Describe the software used to analyze the data in this study.	This study used previously published software: alignments were performed using BLASR (version 1.3.1.124201) and BWA MEM (0.7.12-r1044). Consensus alignments were obtained using kalign (version 2.04). Global alignments of HORs used needle (EMBOSS:6.5.7.0). Repeat characterization was performed using RepeatMasker (Smit, AFA, Hubley, R & Green, P. RepeatMasker Open-4.0. 2013-2015; http://www.repeatmasker.org). Satellite monomers were determined using profile hidden Markov model (HMMER3). Jellyfish (version 2.0.0) was used to characterize k-mers. Illumina read simulations was performed using ART (version 2.5.8). PEAR (version 0.9.0) was used to merge paired read data. Comparative genomic analysis between human and chimpanzee were performed using UCSC Genome Browser liftOver. Additional scripts used in preparing sequences before consensus generation are deposited in GitHub: https://github.com/khmiga/CENY.

For manuscripts utilizing custom algorithms or software that are central to the paper but not yet described in the published literature, software must be made available to editors and reviewers upon request. We strongly encourage code deposition in a community repository (e.g. GitHub). *Nature Methods* guidance for providing algorithms and software for publication provides further information on this topic.

Materials and reagents

Policy information about availability of materials

8. Materials availability

Indicate whether there are restrictions on availability of unique materials or if these materials are only available for distribution by a for-profit company.	No restrictions are associated with this work

9. Antibodies

Describe the antibodies used and how they were validated for use in the system under study (i.e. assay and species).	No antibodies were used in this study. ChIP Seq datasets (using CENPA and CENPC antibodies) were obtained from two previously published studies.

10. Eukaryotic cell lines

a. State the source of each eukaryotic cell line used.	HuRef (Coriell GM25430); A female lymphoblastoid cell line (Coriell GM12708); Pan paniscus (Bonobo) Coriell: AG05253; Pan troglodytes (Common Chimpanzee) Coriell: S006006E. Male gorilla fibroblast cells were provided to Dr. Willard's lab previously by Dr Stephen O'Brien (National Cancer Institute, Frederick, MD).
b. Describe the method of cell line authentication used.	Cell line authentication was determined based information and quality assurance from Coriell biorepository. Source validates cells as described here: https://www.coriell.org/0/pdf/CC_Process_Flow.pdf
c. Report whether the cell lines were tested for mycoplasma contamination.	Cells were tested for mycoplasma contamination by biorepository
d. If any of the cell lines used are listed in the database of commonly misidentified cell lines maintained by ICLAC, provide a scientific rationale for their use.	No commonly mis-identified cell lines were used.

Animals and human research participants

Policy information about studies involving animals; when reporting animal research, follow the ARRIVE guidelines

11. Description of research animals

Provide details on animals and/or animal-derived materials used in the study.	No animals were used in this study.

Policy information about studies involving human research participants

12. Description of human research participants

Describe the covariate-relevant population characteristics of the human research participants.	The study did not involve human research participants.

Nature Biotechnology: doi:10.1038/nbt.4109

nature research

Corresponding author(s): Karen H Miga

☐ Initial submission ☐ Revised version ☒ Final submission

ChIP-seq Reporting Summary

Form fields will expand as needed. Please do not leave fields blank.

Data deposition

1. For all ChIP-seq data:

☒ a. Confirm that both raw and final processed data have been deposited in a public database such as GEO.

☒ b. Confirm that you have deposited or provided access to graph files (e.g. BED files) for the called peaks.

2. Provide all necessary reviewer access links. *The entry may remain private before publication.*	The Centromere protein A (CENP-A) ChIP-seq data used in this study have been previously published, GEO Accession: GSE45497 and GSE60951. Enrichment files (k-mer track and bed file are provided as NBT_SupplementaryDara2.txt and NBT_SupplementaryData3.bed
3. Provide a list of all files available in the database submission.	NBT_SupplementaryDara2.txt<br>NBT_SupplementaryData3.bed
4. If available, provide a link to an anonymized genome browser session (e.g. UCSC).	Genome browser session is not available

Methodological details

5. Describe the experimental replicates.	This is not applicable to this study. ChIP seq data used is previously published and described (PMID: 23230266 & 25927077)
6. Describe the sequencing depth for each experiment.	This is not applicable to this study. ChIP seq data used is previously published and described (PMID: 23230266 & 25927077)
7. Describe the antibodies used for the ChIP-seq experiments.	Anti-CENP-A (Abcam cat #Ab13939)<br>anti-CENP-C (Abcam cat # 33034); as described in PMID: 23230266 & 25927077.
8. Describe the peak calling parameters.	Enrichment was determined as the ratio of the normalized frequency of ChIP-seq data (Anti-CENP-A (Abcam cat #Ab1393 or CENP-C) relative to input control
9. Describe the methods used to ensure data quality.	This is not applicable to this study. ChIP-seq data used is previously published and described (PMID: 23230266 & 25927077)
10. Describe the software used to collect and analyze the ChIP-seq data.	Jellyfish (version 2.0.0; Marçais & Kingsford(2011)) to determine normalized enrichment values

June 2017

Nature Biotechnology: doi:10.1038/nbt.4109
