# Supplementary material for: Linear assembly of a human centromere on the Y chromosome
Source: Nat Biotechnol. 2018 Mar 19;36(4):321–3. doi: 10.1038/nbt.4109 (PMC5886786; doi:10.1038/nbt.4109)
Supplement: Supplementary Table 1 — Throughput for each of the BAC runs. (PDF 149 kb) [file 41587_2018_BFnbt4109_MOESM24_ESM.pdf]

Supplementary Table 1: Throughput for each of the BAC runs.

BAC ID	Type	# reads	Yield (Mb)		Selected Full-length Range (kb)	# reads	Yield (Mb)	Consensus length (bp)
108I14	Control Yp-arm (Yp11.2)	4966	220		75-85	1686	135	80653
531P03	DYZ3 array; unknown order	19620	174		155-175	251	41	165830
808M02	DYZ3 array; unknown order	21936	87		140-180	144	22	149351
1226J10	Yq-arm+HSATIII	23137	716		150-170	689	111	161191
909C13	DYZ3 array; unknown order	24710	969		160-180	400	69	175340
744B15	DYZ3 array; unknown order	348	24		180-200	51	10	194752
890C20	DYZ3 array; unknown order	1221	76		180-195	118	22	190388
648J16	DYZ3 array; unknown order	5485	217		190-200	200	39	198027
718M18	DYZ3 array; unknown order	34354	1239		200-220	525	110	210001
482A22	Control (Xq24)	3437	177		150-200	370	62	170373

SUPPLEMENTARY REFERENCES:

1. Tyler-Smith, C. & Brown, W. R. Structure of the major block of alphoid satellite DNA on the human Y chromosome. *J. Mol. Biol.* **195,** 457–470 (1987).
2. Tyler-Smith, C. Structure of repeated sequences in the centromeric region of the human Y chromosome. *Development* **101 Suppl,** 93–100 (1987).
3. Waye, J. S. & Willard, H. F. Nucleotide sequence heterogeneity of alpha satellite repetitive DNA: a survey of alphoid sequences from different human chromosomes. *Nucleic Acids Res.* **15,** 7549–7569 (1987).

4. Politi, V. *et al.* CENP-C binds the alpha-satellite DNA in vivo at specific centromere domains. *J. Cell Sci.* **115,** 2317–2327 (2002).

5. Warburton, P. E. *et al.* Immunolocalization of CENP-A suggests a distinct nucleosome structure at the inner kinetochore plate of active centromeres. *Curr. Biol.* **7,** 901–904 (1997).

6. Black, B. E. & Cleveland, D. W. Epigenetic centromere propagation and the nature of CENP-a nucleosomes. *Cell* **144,** 471–479 (2011).

7. Levy, S. *et al.* The diploid genome sequence of an individual human. *PLoS Biol.* **5,** e254 (2007).

8. Miga, K. H. *et al.* Centromere reference models for human chromosomes X and Y satellite arrays. *Genome Res.* **24,** 697–707 (2014).

9. Henikoff, J. G., Thakur, J., Kasinathan, S. & Henikoff, S. A unique chromatin complex occupies young α-satellite arrays of human centromeres. *Sci Adv* **1,** (2015).

10. Hayden, K. E. *et al.* Sequences associated with centromere competency in the human genome. *Mol. Cell. Biol.* **33,** 763–772 (2013).
